# MCP mediated active targeting calcium phosphate hybrid nanoparticles for the treatment of orthotopic drug-resistant colon cancer

**DOI:** 10.1186/s12951-021-01115-9

**Published:** 2021-11-17

**Authors:** Shaobo Bai, Yang Sun, Ying Cheng, Weiliang Ye, Chenchao Jiang, Miao Liu, Qifeng Ji, Bangle Zhang, Qibing Mei, Daozhou Liu, Siyuan Zhou

**Affiliations:** 1 Department of Pharmaceutics, School of Pharmacy, Air Force Medical University, Changle West Road 169, Xi’an, 710032 Shaanxi China; 2 Key Laboratory of Gastrointestinal Pharmacology of Chinese Materia Medica of the State Administration of Traditional Chinese Medicine, Department of Pharmacology, School of Pharmacy, Air Force Medical University, Xi’an, 710032 China

**Keywords:** Paris saponin VII, Modified citrus pectin, Drug-resistant colon cancer, Colon cancer targeting drug delivery system, Calcium phosphate nanoparticles

## Abstract

**Background:**

Colon cancer is a most common malignant cancer in digestive system, and it is prone to develop resistance to the commonly used chemotherapy drugs, leading to local recurrence and metastasis. Paris saponin VII (PSVII) could not only inhibit the proliferation of colon cancer cells but also effectively induce apoptosis of drug-resistant colon cancer cells and reduce the metastasis of drug-resistant colon cancer cells as well. However, PSVII was insoluble in water and fat. It displayed no selective distribution in body and could cause severe hemolysis. Herein, colon cancer targeting calcium phosphate nanoparticles were developed to carry PSVII to treat drug-resistant colon cancer.

**Results:**

PSVII carboxymethyl-β-cyclodextrin inclusion compound was successfully encapsulated in colon cancer targeting calcium phosphate nanoparticles (PSVII@MCP-CaP) by using modified citrus pectin as stabilizer agent and colon cancer cell targeting moiety. PSVII@MCP-CaP significantly reduced the hemolysis of PSVII. Moreover, by specific accumulating in orthotopic drug-resistant colon cancer tissue, PSVII@MCP-CaP markedly inhibited the growth of orthotopic drug-resistant colon cancer in nude mice. PSVII@MCP-CaP promoted the apoptosis of drug-resistant colon cancer cells through mitochondria-mediated apoptosis pathway. Moreover, PSVII@MCP-CaP significantly inhibited the invasion and migration of drug-resistant colon cancer cells by increasing E-cadherin protein expression and reducing N-cadherin and MMP-9 protein expression.

**Conclusion:**

PSVII@MCP-CaP has great potential in the treatment of drug-resistant colon cancer. This study also explores a new method to prepare active targeting calcium phosphate nanoparticles loaded with a fat and water insoluble compound in water.

**Graphical Abstract:**

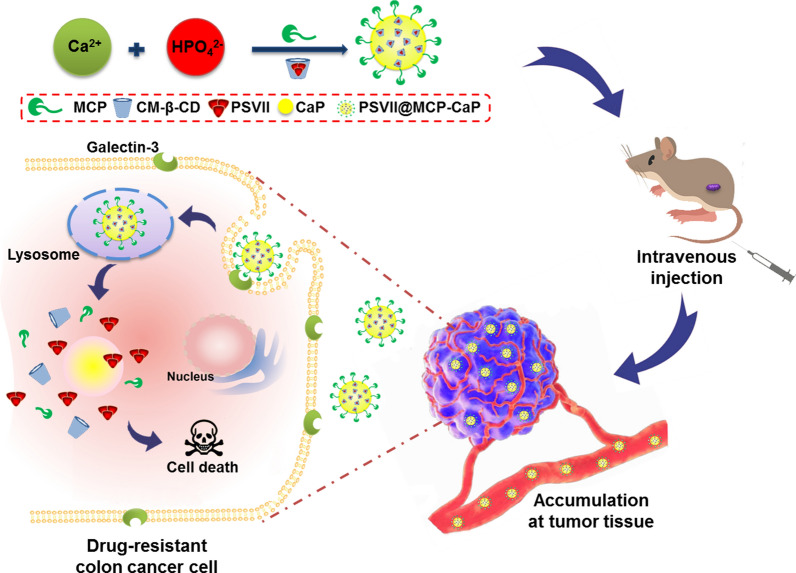

**Supplementary Information:**

The online version contains supplementary material available at 10.1186/s12951-021-01115-9.

## Background

Colon cancer is a most common malignant cancer in digestive system, which is a serious threat to human health. Global cancer statistics results show that colon cancer ranks third in morbidity and second in mortality, and it causes more than 900,000 deaths each year [1]. At present, the treatment method for colon cancer includes surgical resection, radiotherapy, chemotherapy and their combined application, among which chemotherapy plays an important role in treatment of colon cancer [2, 3]. The purpose of chemotherapy is to eliminate the small lesions that can’t be removed by surgery. At the same time, chemotherapy is also used to reduce local recurrence and metastasis of colon cancer, and finally improve the survival rate. With the use of oxaliplatin and irinotecan, chemotherapy has significantly improved the survival time of colon cancer patients. However, colon cancer cell is prone to develop resistance to commonly used chemotherapy drugs, leading to local recurrence and metastasis, which is the main cause of death in colon cancer patients at present [4]. Therefore, it is an urgent need to find new chemotherapy drugs to treat drug-resistant colon cancer.

In our previous studies, we found that Paris saponin VII (PSVII) could not only inhibit the proliferation of colon cancer cells but also effectively induce apoptosis of drug-resistant colon cancer cells and inhibit the metastasis of drug-resistant colon cancer cells [5]. However, PSVII was insoluble in water and fat. It displayed no selective distribution in body and caused severe hemolysis [6]. These disadvantages limited its application in colon cancer treatment. Thus, construction of an appropriate drug delivery system to deliver PSVII to drug-resistant colon cancer cells while reducing its hemolysis effect is the key point for PSVII to inhibit the growth of drug-resistant colon cancer in vivo.

Calcium phosphate nanoparticle (CaP nanoparticles) is an excellent drug delivery system with good safety, biocompatibility and biodegradability [7–9]. CaP nanoparticles, namely amorphous calcium phosphate (ACP), can be formed by Ca^2+^ and PO_4_^3−^ in aqueous solution at room temperature [10]. ACP is metastable and easy to crystallize into calcium phosphate crystals, which significantly reduces its drug loading capacity and greatly limits its application in drug delivery. It was found that some special materials containing carboxyl group and phosphate group, which can bind with Ca^2+^, could increase the stability of CaP nanoparticles [11–13]. We found that carboxymethyl-β-cyclodextrin (CM-β-CD) could effectively encapsulate PSVII to form CM-β-CD inclusion compound (PSVII@CM-β-CD), which markedly increased the solubility of PSVII in water. Moreover, CM-β-CD is able to bind with Ca^2+^ [14]. In the process of preparing CaP nanoparticles in water, PSVII@CM-β-CD could not only stabilize CaP nanoparticles but also realize the embedding of PSVII. However, CaP nanoparticles exhibited low uptake efficiency by cancer cells and no active targeting effect on drug-resistant colon cancer cell, which seriously affects the therapeutic effect of PSVII-encapsulated CaP nanoparticles to colon cancer in vivo. Recently, we found that galectin-3 (Gal-3) highly expressed on the cell membrane of drug-resistant colon cancer cells, and modified citrus pectin (MCP) could selectively bind with Gal-3 [15–17]. Moreover, MCP contains a large number of carboxyl groups, which are able to combine with Ca^2+^ to reduce the recrystallization of ACP and improve the stability of CaP nanoparticles.

**Scheme 1:**
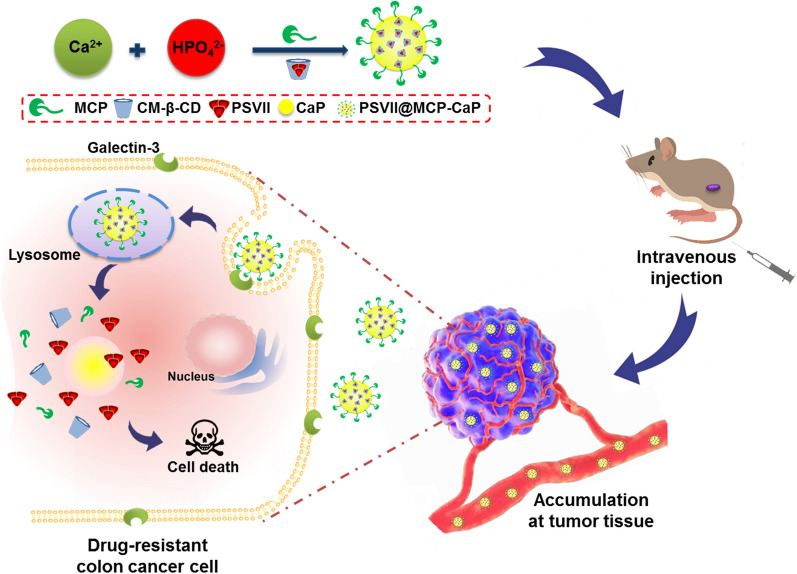
Schematic illustration of the preparation and cellular uptake of PSVII@MCP-CaP

In this study, by using PSVII as an anti-cancer drug, CM-β-CD as the encapsulating material of PSVII, MCP as a targeting ligand of drug-resistant colon cancer cells, PSVII@CM-β-CD and MCP also as stabilizers of CaP nanoparticles, colon cancer targeting CaP nanoparticles loading with PSVII (PSVII@MCP-CaP) was prepared (Scheme 1). PSVII@MCP-CaP was supposed to decrease the hemolysis effect of PSVII. At the same time, PSVII@MCP-CaP increased the distribution of PSVII in orthotopic drug-resistant colon cancer tissues and improved the inhibitory effect of PSVII on orthotopic drug-resistant colon cancer.

## Materials and methods

### Materials

PSVII was purchased from Shanghai Yuanye Biotechnology Co. (Shanghai, China). Modified citrus pectin (MCP) was obtained from Centrax International (USA). Oxaliplatin (L-OHP) and 5-fluorouracil (5-Fu) were purchased from Energy Chemical (Shanghai, China). Carboxymethyl-β-cyclodextrin (CM-β-CD) was obtained from Shandong Binzhou Zhiyuan Biotechnology Co. (Binzhou, China). Paraformaldehyde, 4ʹ,6-diamidino-2-phenylindole dihydrochloride (DAPI) and 3-(4,5-dimethylthiazol-2-yl)-2,5-diphenyltetrazolium bromide (MTT) were purchased from Xi’an Kehao Biotechnology Co. (Xi’an, China). RPMI-1640 cell culture medium and fetal bovine serum (FBS) were obtained from HyClone (USA). Penicillin–streptomycin mixture and trypsin/EDTA solution were purchased from Solarbio (Beijing, China). Antibodies were obtained from Abcam (USA). The SDS-PAGE was purchased from Beyotime (Shanghai, China). HCT116 cell, HT-29 cell, SW620 cell and NCM460 cell were bought from ATCC (USA). 5-Fu-resistant HCT116 cell (HCT116/Fu cell) and L-OHP-resistant HCT116 cell (HCT116/L cell) were obtained from Shanghai Zhewen Biotechnology Co. (Shanghai, China). Nude mice and SD rats were purchased from the Experimental Animal Center of Air Force Medical University (Xi’an, China).

### Gal-3 expression in normal colon tissue and orthotopic drug-resistant colon cancer tissue

The normal colon tissue and orthotopic drug-resistant colon cancer tissue were fixed with 4% paraformaldehyde solution for 24 h, and then they were sliced after being embedded in paraffin. The sections were incubated with Gal-3 antibody for 1 h, and then sections were incubated with PE-labeled secondary antibody for 1 h. Gal-3 in normal colon tissue and orthotopic drug-resistant colon cancer tissue were observed by laser scanning confocal microscopy (LSCM, FV3000, Olypus, Japan).

### Detection of Gal-3 expression in normal colon epithelial cell and colon cancer cell

Normal colon epithelial cell (NCM460 cell) and colon cancer cell (including HCT116 cell, HCT116/F cell, HCT116/L cell, HT-29 cell and SW620 cell) in the logarithmic growth phase were planted in culture dish containing 8 mL culture medium at density of 1 × 10^6^ cells/dish. After being cultured for 24 h, the cells were collected and lysed with cell lysate in an ice bath for 30 min. The cell lysate was centrifuged for 15 min (13,200×*g*), and the supernatant was collected. Western blot was used to detect Gal-3 in the supernatant.

### Localization of Gal-3 in normal colon epithelial cell and colon cancer cell

NCM460 cell, HCT116 cell, HCT116/F cell, HCT116/L cell, HT-29 cell and SW620 cell in logarithmic growth phase was respectively inoculated in cover glass-containing 24-well plate (500 μL/well) with density of 1 × 10^4^ cells /mL and was cultured for 24 h. After being rinsed with PBS, the cover glass was fixed with 4% paraformaldehyde solution for 15 min, and then sealed with 5% BSA solution for 30 min. Next, Gal-3 primary antibody was added and incubated overnight at 4 ℃. The cover glass was incubated with Cy5-labeled secondary antibody for 1 h at room temperature. Next, DIO membrane dye solution was incubated with cover glass at room temperature for 30 min. The cover glasses were cleaned with PBS, and then they were incubated with DAPI solution at room temperature for 20 min. Finally, cover glasses were sealed with anti-fluorescence quenching sealing solution, and the localization of Gal-3 in colon cancer cells was observed under LSCM.

### Preparation and characterization of PSVII@MCP-CaP

PSVII (8.0 mg) and CM-β-CD (32.0 mg) were dissolved into 4.0 mL deionized water and stirred for 4 h. MCP (60 mg) was added into above solution and stirred at room temperature for 10 min. Then, 500 µL of CaCl_2_ and (NH_4_)_2_HPO_4_ solution were slowly added and stirred at 80 ℃ for 1 h. 40 mL deionized water was added into the mixture solution, and ultrasound was applied for 3 min. Next, 0.45 μm microporous filtration membrane was used to filter the mixture solution. Finally, PSVII@MCP-CaP powder was obtained after lyophilization of filtrate. By using Cy7.5 labelled PSVII (Cy7.5-PSVII), Cy7.5-PSVII@MCP-CaP was prepared with the same method in the preparation of PSVII@MCP-CaP. Sulfur labeled PSVII was obtained by modifying PSVII with 3-mercaptopropionic acid. Then, sulfur labeled PSVII was encapsulated in CaP nanoparticle by using the same method in the preparation of PSVII@MCP-CaP. Blank nanoparticle (without PSVII, @MCP-CaP) was prepared by using the same method in the preparation of PSVII@MCP-CaP. Particle size, stability and zeta potential of PSVII@MCP-CaP were measured by zeta potential and nanoparticle analyzer (Delsa Nano C, Beckman, USA).

The appearance of PSVII@MCP-CaP was observed by transmission electron microscope (TEM, Tecnai G2 Spirit, FEI, USA). The encapsulation of sulfur labeled PSVII in CaP nanoparticle was investigated by element mapping analysis. The drug loading and drug release characteristics of PSVII@MCP-CaP were determined by high performance liquid chromatograph (HPLC, Waters 2695/2996, USA). Briefly, PSVII@MCP-CaP (30 mg) was dispersed in PBS (pH5.0 and pH7.4) at concentration of 6 mg/mL. The PSVII@MCP-CaP solution was transferred into a dialysis bag with an interception molecular weight of 5000 Da. Then dialysis bag was immerged in the same release medium (60 mL) as that in dialysis bag. 0.5 mL of the release medium out of dialysis bag was taken out at different time point, and 0.5 mL of the corresponding fresh release medium was added into the solution out of dialysis bag. The concentration of PSVII in release medium was determined by HPLC, and the cumulative release amount was calculated. The drug release curve was plotted.

Waters Symmetry C_18_ (50 mm × 2.1 mm, 5 μm) was used as analytic column. Acetonitrile and water (v:v = 50:50) was used as mobile phase. The flow rate was 1 mL/min. The column temperature was 25 ℃. The detection wavelength was 203 nm. The equation of the standard curve was *Y* = 136.66*X* + 97.13, R^2^ = 0.9999.

### Hemolysis analysis

10 mL rat whole blood was diluted with 100 mL normal saline. The mixture was slightly shaken and centrifuged for 5 min (3000 rpm). Red blood cells were collected and washed with normal saline for 3 times. Red blood cells were collected. 1% red blood cell suspension was prepared with normal saline as the dispersion medium. Then, 100 μL @MCP-CaP solution, PSVII solution, PSVII@MCP-CaP solution and normal saline were added into 5 mL red blood cell suspension, respectively. The mixture was incubated in 37 ℃ water bath for 40 min. The mixture was centrifuged for 5 min at 4 ℃ (3000 rpm), then supernatant and red blood cells were separated. (1) Red blood cells were dispersed into 2 mL glutaraldehyde solution and fixed for 2 h. Then it was dehydrated by successively using 10%, 20%, 40%, 80% and 90% alcohol for 10 min each time. The dehydrated red blood cells were dispersed into anhydrous ethanol. Finally, the red blood cells were dropped onto the slide and dried at room temperature for 2 h. The morphology of red blood cells was observed by scanning electron microscope (SEM, Hitachi, S4800, Japan). (2) The absorbance of the supernatant at 414 nm was measured with an ultraviolet spectrophotometer, and the hemolysis rate (HR) was calculated. HR (%) = (absorbance of sample − absorbance of negative control)/(absorbance of positive control − absorbance of negative control) × 100%.

### X-ray photoelectron spectroscopy analysis and thermogravimetric analysis

The surface elements of PSVII@MCP-CaP were determined by X-ray photoelectron spectrometer (ESCALAB250Xi, Thermo Fisher Scientific, USA). The scanning times were 10. The excitation source was Al Ka X-ray, and the analysis point area was 650 µm. The analyzer mode was CAE. The band pass energy was 20.0 eV, and the analysis energy step was 0.05 eV.

Calcium phosphate powder (CaP powder, 5 mg), @MCP-CaP (5 mg), PSVII@MCP-CaP (5 mg) and MCP (5 mg) were put into crucible, respectively. DTG thermogravimetric analyzer (DSC-03, PerkinElmer, USA) was used for comprehensive thermal analysis. The scanning temperature was 0–500 °C. The protective gas was nitrogen, and heating rate was 10 °C/min.

### Cellular uptake experiment

HCT116/L cells in logarithmic growth phase were inoculated into 24-well plate (500 μL/well) with cover glass at density of 1 × 10^4^ cells/mL and cultured for 24 h. The culture medium was replaced with serum-free fresh medium containing Cy7.5-PSVII@MCP-CaP, and the amount of PSVII in per well was 2 μmol and 5 μmol. After cells were cultured for 0.5 h, 2 h and 4 h, the cover glass was fixed with 4% paraformaldehyde solution for 15 min. Next, the cover glass was stained by 1 mL DAPI solution (600 ng/mL) for 15 min. The cover glasses were cleaned with PBS and sealed with glycerin. Finally, the fluorescence in HCT116/L cells was observed by LSCM.

HCT116/L cells in logarithmic growth phase were seeded into 24 well plates (500 μL/well) with cover glass at density of 1 × 10^4^ cells/mL and cultured for 24 h. The culture medium was replaced with serum-free fresh culture medium containing MCP (12.5, 6.25, 3.125 μg/mL) and cultured for 1 h. Then, Cy7.5-PSVII@MCP-CaP was added into cell culture medium. After cells were cultured for 4 h, the cover glass was fixed with 4% paraformaldehyde solution for 15 min. Next, 1 mL DAPI solution (600 ng/mL) was used to stain cell nucleus on cover glass for 15 min. The cover glass was cleaned with PBS and sealed with glycerin. Finally, the fluorescence in HCT116/L cells was observed by LSCM.

HCT116 cells and HCT116/L cells in logarithmic growth phase were inoculated in 24-well plates containing agarose gel (cell density was 1 × 10^3^ cells/well), and culture medium was replaced by fresh culture medium every other day. When the diameter of cell spheres reached 100–200 μm, the cell culture medium was replaced with fresh cell culture medium containing Cy7.5-PSVII@MCP-CaP (equivalent PSVII concentration was 1.0 μmol/L). After incubation for 2 h, the cell culture medium was removed, and 4% paraformaldehyde solution was used to fix cell spheres. The distribution of fluorescence in colon cancer cell spheres was observed by LSCM.

HCT116/L cells in logarithmic growth phase were planted into 24 well plates (500 μL/well) with cover glass at density of 1 × 10^4^ cells/mL and cultured for 24 h. After pretreatment with sucrose (clathrin endocytosis inhibitor), 2-deoxy-d-glucose (ATP depletion agent), colchicine (macropinocytosis inhibitor) and methyl-β-cyclodextrin (caveolin endocytosis inhibitor) for 1 h, fresh cell culture medium containing Cy7.5-PSVII@MCP-CaP was added and cultured for 4 h. The cover glass was fixed with 4% paraformaldehyde solution for 15 min, and then DAPI solution (600 ng/mL) was used to stain cell nucleus on cover glass for 15 min. Finally, the cover glass was cleaned with PBS and sealed with glycerin. The fluorescence in HCT116/L cells was observed by LSCM.

### Cytotoxicity test

MTT assay was used to detect the cytotoxicity of PSVII@MCP-CaP on colon cancer cells. HCT116 cell, HCT116/L cell, HT-29 cell and SW620 cell at logarithmic growth phase were inoculated into 96-well plate at density of 5 × 10^4^ cells/mL (200 μL/well) and cultured for 24 h. The cell culture medium was replaced with 200 μL fresh cell culture medium containing PSVII, L-OHP and PSVII@MCP-CaP, and cells were cultured for 24 h. MTT solution (20 μL, 5 mg/mL) was added into each well, and cells were incubated for 4 h. After removing cell culture medium, 150 μL DMSO was added into each well. The absorbance of each well was measured at 490 nm. The inhibition rate was calculated as follows: inhibition rate % = [1 − (absorbance of drug treatment group − absorbance of the blank group)/(absorbance of the control group − absorbance of the blank group)] × 100%.

Live/dead cell staining was also used to investigate the cytotoxicity of PSVII@MCP-CaP on colon cancer cells. HCT116 cell, HCT116/L cell, HT-29 cell and SW620 cell at logarithmic growth phase were inoculated into 6-well plate at density of 5 × 10^5^ cells/mL (2 mL/well) and cultured for 24 h. The cell culture medium was replaced with fresh cell culture medium containing PSVII, L-OHP and PSVII@MCP-CaP, the drug concentration was 3 μmol/L. Cells were cultured for 48 h. The adherent cells were prepared into suspension and washed twice with assay buffer. Then 1 mL live/dead staining solution was added to disperse the cells, and the cells were incubated at 37 ℃ for 20 min. The cells were washed with assay buffer for 2 times, and then 0.5 mL PBS was added to disperse the cells. Finally, the living and dead cells was observed under fluorescence microscope.

### Clone formation experiment

HCT116 cells and HCT116/L cells at logarithmic growth phase were inoculated into 6-well plates at density of 200 cells/well and cultured for 24 h. The cell culture medium was replaced with 2 mL of fresh cell culture medium containing PSVII and PSVII@MCP-CaP (equivalent PSVII concentration was 0.5 and 1 μmol/L). After incubation for 24 h, the cell culture medium was replaced with fresh drug-free cell culture medium. The cells were observed daily, and cell culture medium was replaced with fresh drug-free cell culture medium every other day. The culture was terminated when multiple clones were visible. The supernatant was removed, and cell clones were fixed with 4% paraformaldehyde solution for 15 min. Then 0.1% crystal violet was added to stain cell clones for 15 min. After that, PBS was used to wash cell clones. After drying of cell clones, the number of clones was counted under microscope, and the clone formation rate was calculated. Clone formation rate = (number of clones/number of inoculated cells) × 100%.

### Migration experiment

HCT116 cell, HCT116/L cell, HT-29 cell and SW620 cell in logarithmic growth phase were inoculated into donor chamber of 24-well transwell chamber at density of 4 × 10^4^ cells/well, and 600 μL RPMI-1640 culture medium containing 30% fetal bovine serum was added into recipient chamber. After cells were cultured for 6 h, cell culture medium containing PSVII, PSVII@MCP-CaP (equivalent PSVII concentration was 0.5 and 1 μmol/L) was added into donor chamber, respectively. After cells were cultured for 24 h, transwell chambers were taken out and fixed with 4% paraformaldehyde solution for 15 min. Then, 0.1% crystal violet was added into transwell chambers to stain migrated cells for 10 min. After being rinsed with PBS for 3 times, transwell chamber was placed under a microscope and photographed. Next, 800 μL of 33% glacial acetic acid solution was added into the transwell chamber, and the absorbance of glacial acetic acid solution at 570 nm was measured. Finally, the relative migration rate was calculated. The relative migration rate (%) = (absorbance of drug treatment group/absorbance of the control group) × 100%.

### Invasion experiment

Matrigel was diluted with RPMI-1640 medium precooled at 4 ℃ (cell culture medium:matrigel = 9:1) on an ice bath. 100 μL diluted matrigel was added into donor chamber of 24-well plate and incubated at 37 ℃ for 2 h to coagitate matrigel fully. HCT116 cell, HCT116/L cell, HT-29 cell and SW620 cell in logarithmic growth phase were seeded into donor chamber of 24-well plate containing matrigel at density of 4 × 10^4^ cells/well. 400 μL RPMI-1640 medium containing 30% fetal bovine serum was added into recipient chamber. After cells were cultured for 6 h, cell culture medium containing PSVII, PSVII@MCP-CaP (equivalent PSVII concentration was 0.5 and 1 μmol/L) were added into donor chamber, respectively. After being cultured for 24 h, transwell chambers were taken out and fixed with 4% paraformaldehyde solution for 15 min. Then, 0.1% crystal violet was added into transwell chambers to stain invaded cell nucleus for 10 min. After being rinsed with PBS for 3 times, transwell chamber was placed under a microscope and photographed. Next, 800 μL of 33% glacial acetic acid solution was added into the transwell chamber, and the absorbance of glacial acetic acid solution at 570 nm was measured. Finally, the relative invasion rate was calculated. The relative invasion rate (%) = (absorbance of drug treatment group/absorbance of the control group) × 100%.

### 3D drug-resistant colon cancer cell sphere experiment

HCT116 cells and HCT116/L cells at logarithmic growth phase were inoculated into 24-well plates containing agarose gel (500 μL/well) at density of 1 × 10^3^ cells/well. The cell culture medium was replaced by fresh cell culture medium every other day. After incubation for 2 weeks, the formation and growth of colon cancer cell spheres were observed. When the cell sphere diameter was about 200 μm, 500 μL fresh cell culture medium containing PSVII (1.0 μmol/L) and PSVII@MCP-CaP (equivalent PSVII concentration was 1 μmol/L) were added, respectively. After incubation for 24 h, cell culture medium was replaced with fresh drug-free cell culture medium. On day 1, 3, 5, 7 and 9 after drug treatment, the growth of cell spheres was observed by microscope. The cell sphere volume was calculated as V (volume) = (long diameter × short diameter^2^)/2.

### Apoptosis-related proteins and invasion-related proteins in drug-resistant colon cancer cells

HCT116 cell, HCT116/L cell, HT-29 cell and SW620 cell in logarithmic growth phase were inoculated in 6-well plates at density of 1 × 10^6^ cells/mL and cultured for 24 h. The cell culture medium was replaced with 2 mL fresh cell culture medium containing PSVII (0.5 and 1 μmol/L) and PSVII@MCP-CaP (equivalent PSVII concentration was 0.5 and 1 μmol/L), respectively. After incubation for 24 h, the total proteins of cells were extracted. The apoptosis-related proteins and invasion-related proteins in cell lysates were detected by western blot.

### Orthotopic drug-resistant colon cancer model

Luciferase labeled oxaliplatin-resistant human colon cancer cells (HCT116/L-Luc) were re-suspended in serum-free medium and inoculated subcutaneously in the back of 5-week-old nude mice (200 μL/mouse, 2 × 10^7^ cells/mL). When the diameter of subcutaneous cancer was about 1–1.5 cm, the nude mice were anesthetized, and cancer tissue was stripped from the subcutaneous tissue under sterile conditions. The cancer tissue was cut into small pieces with a diameter of 1 mm.

5-week-old nude mice were anesthetized and dissected along the right midline of the abdomen to expose the colon. The serous membrane layer of the colon was cut at the site with abundant blood supply on the surface of the colon (about 1 cm away from the cecum), and then the sterile small curved forceps were used to push the incision inwardly to form a local groove. The pre-cut cancer tissue was placed in the groove, and 2–3 drops of OB biological glue were dropped to fix tumor tissue. The colon was reset, and incision was sutured with sterile silk thread. Three days later, luciferase substrate was intraperitoneally injected to the mice (150 mg/kg), the orthotopic colon cancer growth was observed by in vivo imaging (Caliper IVIS Lumina II, Siemens, Germany).

### Biodistribution of PSVII@MCP-CaP in orthotopic drug-resistant colon cancer nude mice

On the 10th day after orthotopic drug-resistant colon cancer model was successful set up, Cy7.5-PSVII (5 mg/kg) and Cy7.5-PSVII@MCP-CaP (equivalent Cy7.5-PSVII concentration was 5 mg/kg) were injected by the tail vein of nude mice. The brain, heart, liver, spleen, lung, kidney and colon cancer tissue of nude mice were isolated at 12 h and 24 h. The distribution of fluorescence in orthotopic colon cancer tissue and normal organs of nude mice was observed by in vivo bioluminescence imaging. Next, the brain, heart, liver, spleen, lung, kidney and tumor tissues were placed in a glass grinder, and RIPA lysate (200 μL/100 mg tissue) was added. The tissues were thoroughly ground in ice bath. The homogenate was centrifuged at 4 ℃ for 15 min (13,200×*g*) to obtain the supernatant. Finally, fluorescence intensity in supernatant of brain, heart, liver, spleen, lung, kidney and tumor tissue was measured by fluorescence spectrophotometer.

### The inhibitory effect of PSVII@MCP-CaP on the growth of orthotopic drug-resistant colon cancer in nude mice

On the 4th day after orthotopic drug-resistant colon cancer model was set up, luciferase substrate was intraperitoneally injected to mice (150 mg/kg), the orthotopic colon cancer growth was observed by in vivo bioluminescence imaging. The unqualified nude mice were removed, and the remaining orthotopic drug-resistant colon cancer nude mice were divided into four groups. The fluorescence intensity of orthotopic colon cancer tissues in each group showed no significant difference. The cancer-bearing nude mice were given normal saline, oxaliplatin (5 mg/kg), PSVII (5 mg/kg) and PSVII@MCP-CaP (5 mg/kg, equivalent of PSVII) by tail vein injection every 4 days. After 7 times of drug administration, the experiment was terminated. Luciferase substrate was intraperitoneally injected into mice (150 mg/kg), and the growth of orthotopic colon cancer tissue was observed by in vivo imaging. The colon of nude mice was isolated and photographed. The weight and size of colon cancer tissue was measured. Colon cancer tissue, brain, heart, liver, spleen, lung and kidney of nude mice were isolated and fixed with 4% paraformaldehyde solution. H&E staining was used to observe the histological morphology of colon cancer tissue and normal tissue. The apoptosis-related proteins and invasion-related proteins in colon cancer tissue were detected by western blot.

### Statistical analysis

Data are expressed as the mean ± standard deviation (SD). The statistics analysis was performed by using a one-way ANOVA method. *p* < 0.05 was considered statistically significant.

## Results

### Expression of Gal-3 in drug-resistant colon cancer tissue and colon cancer cells

**Fig. 1:**
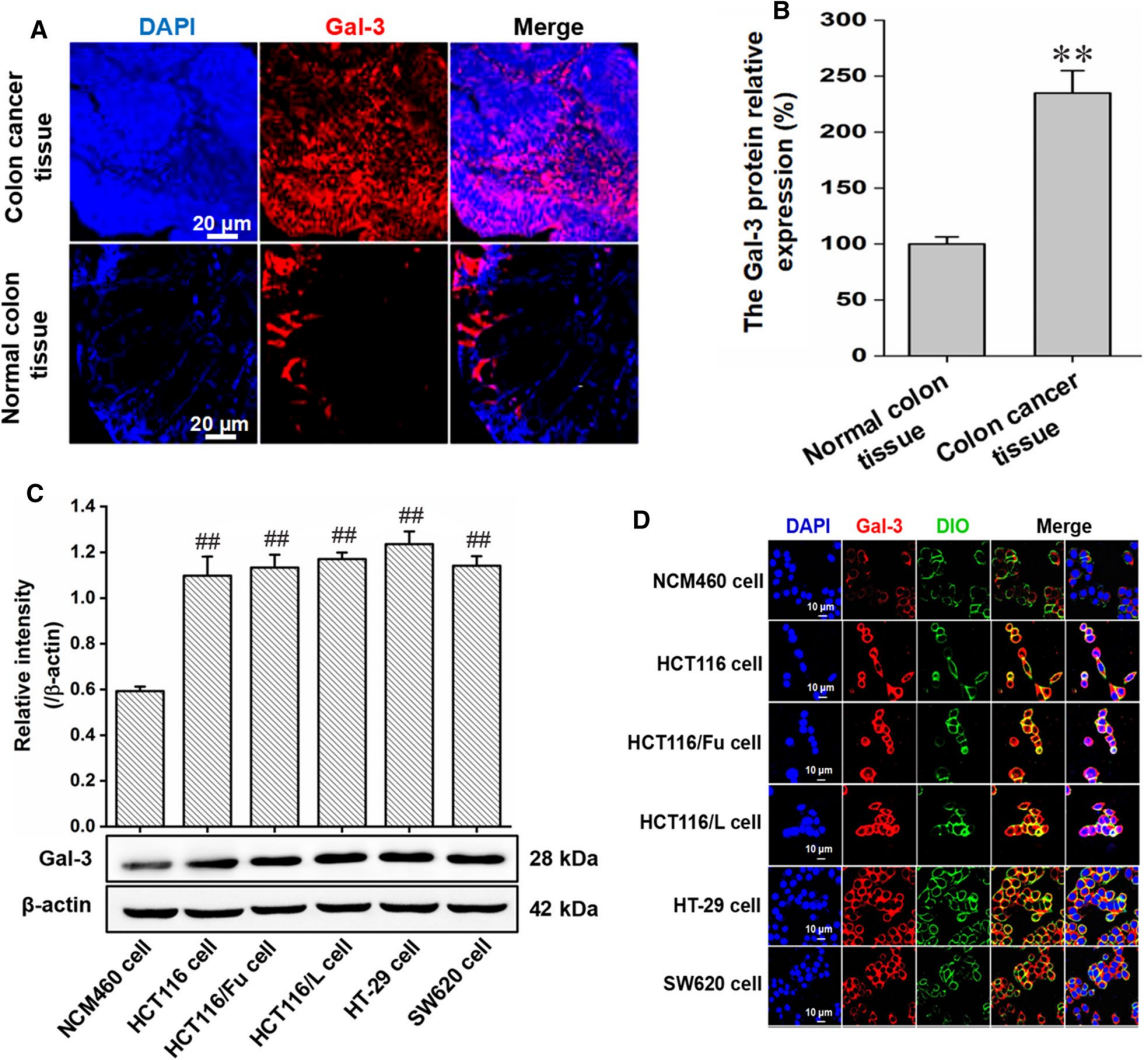
The expression of Gal-3 in vitro and in vivo. **A** Expression of Gal-3 in drug-resistant colon cancer tissue and normal colon tissue of mice. **B** Semi-quantitative statistical results of Gal-3 expression in drug-resistant colon cancer tissue and normal colon tissue. **C** The expression of Gal-3 in NCM460 cell, HCT116 cell, HCT116/Fu cell, HCT116/L cell, HT-29 cell and SW620 cell detected by western blot. **D** The distribution of Gal-3 in NCM460 cell, HCT116 cell, HCT116/Fu cell, HCT116/L cell, HT-29 cell and SW620 cell observed by LSCM. All data are expressed as mean ± SD (n = 3). **p < 0.01 vs normal colon tissue; ^##^*p* < 0.01 vs NCM460 cell

Immunofluorescence staining results showed that a larger amount of Gal-3 was expressed in drug-resistant colon cancer tissue than in normal colon tissue (Fig. 1A, B; Additional file 1: Fig. S1). Western blot results indicated that in comparison with normal colon epithelial cells (NCM460 cells), Gal-3 protein expression was significantly increased in colon cancer cell line such as HCT116 cell, HCT116/Fu cell, HCT116/L cell, HT-29 cell and SW620 cell (Fig. 1C). In addition, LSCM results showed a large amount of red fluorescence localized on the cell membrane of HCT116 cell, HCT116/Fu cell, HCT116/L cell, HT-29 cell and SW620 cell (Fig. 1D), indicating that Gal-3 highly expressed on the cell membrane of colon cancer cell.

### Characterization of PSVII@MCP-CaP

**Fig. 2:**
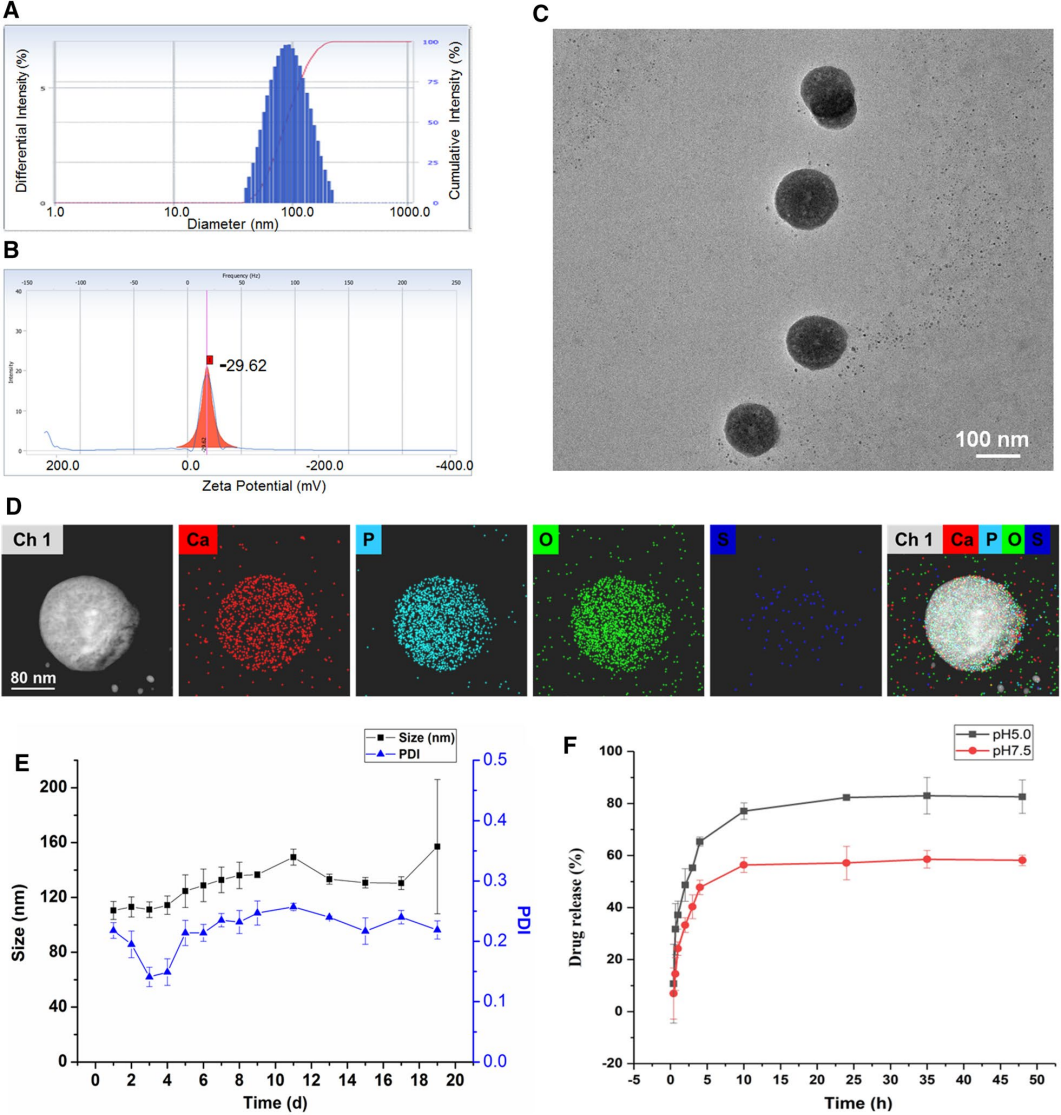
Characterization of PSVII@MCP-CaP. **A** Particle size distribution of PSVII@MCP-CaP. **B** Zeta potential of PSVII@MCP-CaP. **C** TEM image of PSVII@MCP-CaP. **D** Element mapping analysis of MCP-CaP nanoparticles encapsulated with sulfur-labeled PSVII. **E** Stability of PSVII@MCP-CaP in deionized water. **F** In vitro drug release of PSVII@MCP-CaP. All data are expressed as mean ± SD (n = 3)

The average hydration particle size and the average zeta potential of PSVII@MCP-CaP were 104 nm and − 29.62 mV, respectively (Fig. 2A, B). TEM results showed that the appearance of PSVII@MCP-CaP was spherical with good dispersion (Fig. 2C). The X-ray photoelectron spectroscopy (XPS) results indicated that PSVII@MCP-CaP contained calcium, phosphorus, carbon and oxygen (Additional file 1: Fig. S2). The DTG results are showing in Additional file 1: Fig. S3. Pure calcium phosphate powder (CaP powder) had a major weight loss around 160–300 ℃, and the peak of DTG was sharp and narrow. The main weight loss range of MCP was 150–350 ℃. Meanwhile, the main peak of DTG had bifurcation and trailing, and the peak was relatively wide. This may be caused by the absence of a fixed melting point of MCP. PSVII@CM-β-CD inclusion compound mainly loosed weight around 300–450 ℃, and its peak shape was wide. Compared with CaP, @MCP-CaP showed a similar initial temperature of weight loss, but weight loss rate slowed down and weight loss range increased to around 350 ℃. The main peak of DTG widened with tails and the overall percentage of weight loss increased, indicating that CaP nanoparticles had been modified by MCP. Compared with @MCP-CaP, PSVII@MCP-CaP showed obvious double peaks in DTG. The peak position of the big peak was basically consistent with that of PSVII@CM-β-CD, and the peak position of the late small peak was consistent with that of PSVII, which was speculated to be caused by PSVII loading. The above results indicated that PSVII@MCP-CaP contained calcium phosphate, PSVII and MCP. Furthermore, the element mapping analysis showed that sulfur-labeled PSVII mainly located in the interior of MCP-CaP nanoparticles (Fig. 2D). This indicated that PSVII was successfully encapsulated in MCP-CaP nanoparticle.

The stability results showed that particle size and PDI of PSVII@MCP-CaP in aqueous solution did not change significantly within 18 days (Fig. 2E), indicating that PSVII@MCP-CaP was relatively stable within 18 days. Besides, the drug loading of PSVII in PSVII@MCP-CaP was 5.12%, and MCP content in PSVII@MCP-CaP was 5.43%. The encapsulation rate of PSVII was 83.2%. Moreover, PSVII released from PSVII@MCP-CaP displayed a pH-sensitive manner. In pH 7.5 release medium, the cumulative drug release was 40% and 57% within 3 h and 24 h, respectively. In pH 5.0 release medium, the cumulative drug release was 55% and 82% within 3 h and 24 h (Fig. 2F), respectively.

**Fig. 3:**
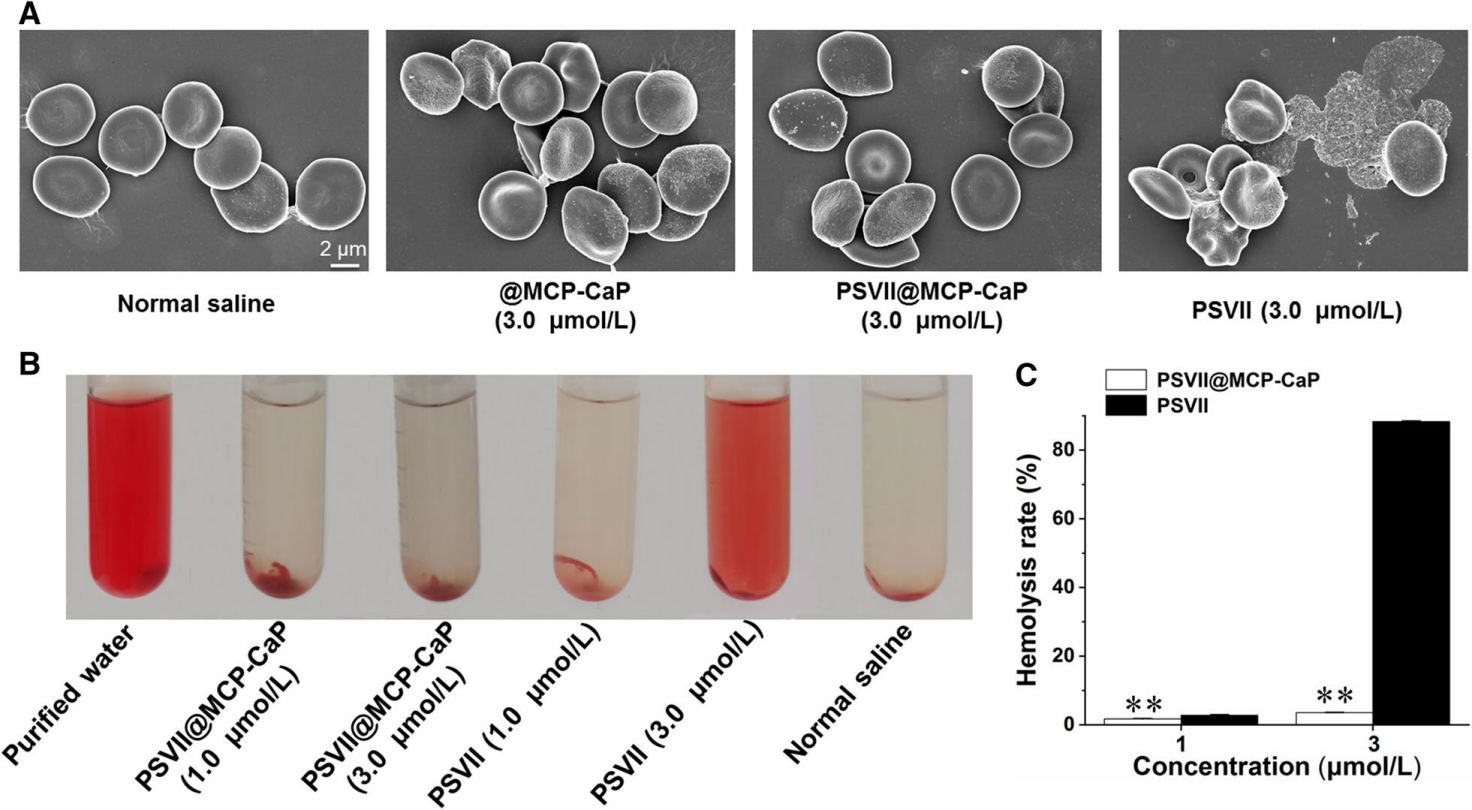
Hemolysis effect of PSVII@MCP-CaP. **A** SEM images of blood red cell after incubation with PSVII and PSVII@MCP-CaP. **B** Hemolysis phenomenon caused by PSVII and PSVII@MCP-CaP. **C** Statistical results of hemolysis rate. All data are expressed as mean ± SD (n = 3). **p < 0.01 vs PSVII

The effect of PSVII@MCP-CaP on morphological characteristics of erythrocyte membrane is showing in Fig. 3A. The erythrocyte membrane in normal saline treated and @MCP-CaP treated group was intact. PSVII@MCP-CaP had little effect on red blood cell, and there was no obvious red blood cell fragment. PSVII obviously destroyed the red blood cell membrane, and there were a large number of red blood cell fragments. The hemolysis effect of PSVII@MCP-CaP is showing in Fig. 3B, C. The results showed that hemolysis effect of PSVII was very strong, and the hemolysis effect PSVII@MCP-CaP was significantly lower in comparison with PSVII. These results demonstrated that PSVII@MCP-CaP markedly reduced damage of erythrocyte membrane caused by PSVII.

### The uptake of PSVII@MCP-CaP by drug-resistant colon cancer cells

**Fig. 4:**
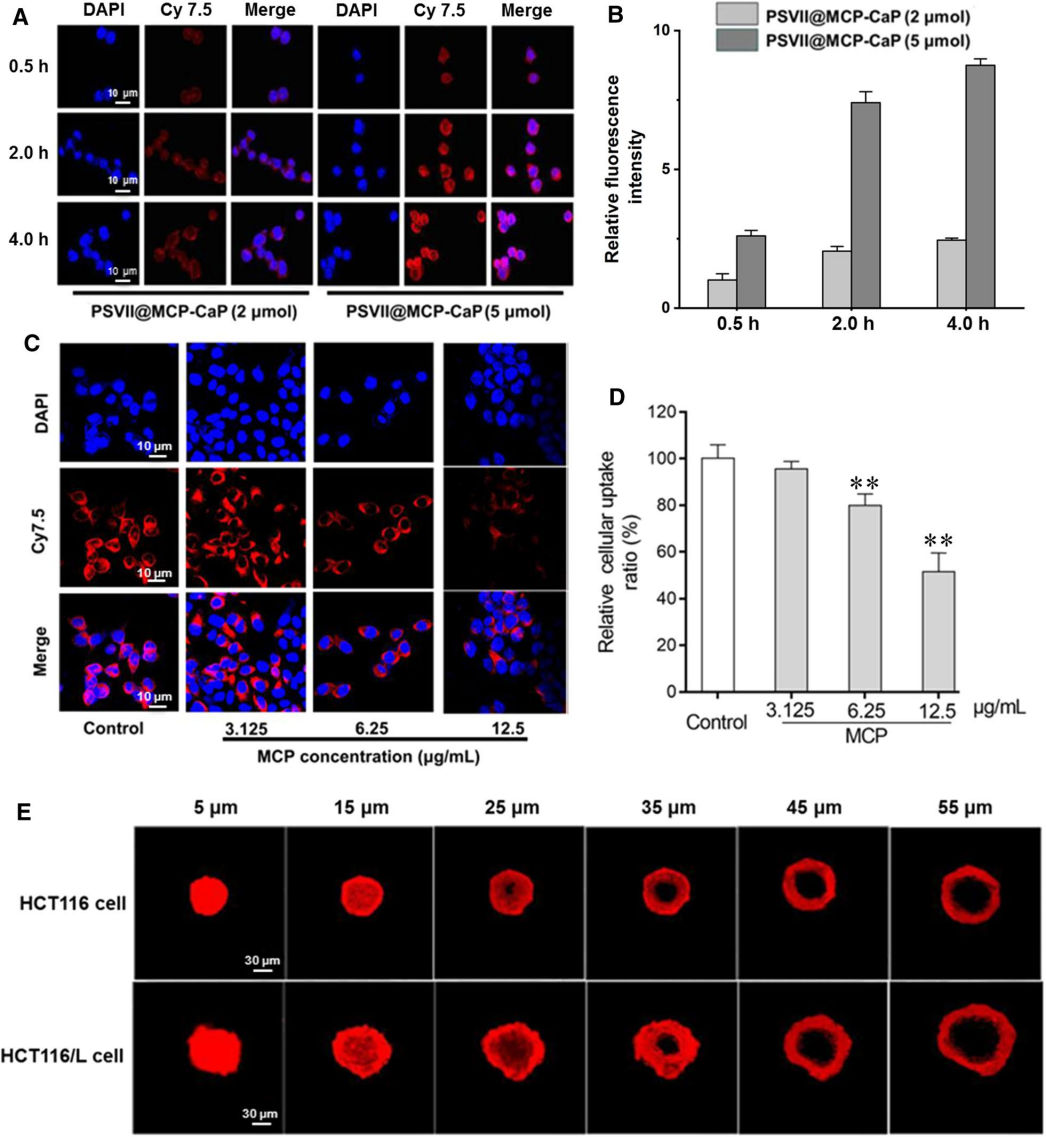
The uptake of PSVII@MCP-CaP by HCT116/L cell and HCT116/L cell spheres. **A** The uptake of PSVII@MCP-CaP by HCT116/L cell. **B** Semi-quantitative statistical results of uptake of PSVII@MCP-CaP by HCT116/L cell. **C** The effect of MCP on the uptake of PSVII@MCP-CaP by HCT116/L cell. **D** Semi-quantitative statistical results of the effect of MCP on the uptake of PSVII@MCP-CaP by HCT116/L cell. **E** Distribution of PSVII@MCP-CaP in 3D HCT116/L cell sphere. All data are expressed as mean ± SD (n = 3). **p < 0.01 vs control

As shown in Fig. 4A, B, with the increase of incubation time and the concentration of PSVII@MCP-CaP, the red fluorescence intensity in HCT116/L cells was gradually enhanced, indicating the uptake of PSVII@MCP-CaP by HCT116/L cells was gradually increased. These results implied that the uptake of PSVII@MCP-CaP by HCT116/L cells exhibited concentration-dependent and time-dependent manner. When HCT116/L cells were co-incubated with MCP for 1 h before adding PSVII@MCP-CaP, the intracellular red fluorescence intensity was significantly reduced. The higher the MCP concentration was, the lower the intensity of intracellular fluorescence became (Fig. 4C, D). This suggested that MCP mediated the uptake of PSVII@MCP-CaP by HCT116/L cells.

After incubating with colon cancer cell spheres for 2 h, PSVII@MCP-CaP penetrated into the deep region of 3D colon cancer cell spheres (Fig. 4E). This provided the basis for the excellent anticancer effect of PSVII@MCP-CaP in vivo.

The uptake mechanism of PSVII@MCP-CaP by HCT116/L was further investigated by using LSCM. The results showed that 2-deoxy-d-glucose (2-DG), colchicine and sucrose significantly decreased the uptake of PSVII@MCP-CaP by HCT116/L cells. Methyl-β-cyclodexin (M-β-CD) displayed no significant effect on the uptake of PSVII@MCP-CaP by HCT116/L cells (Additional file 1: Fig. S4). These results demonstrated that HCT116/L cells took up PSVII@MCP-CaP mainly through clathrin mediated endocytosis.

### Effects of PSVII@MCP-CaP on proliferation, clone formation, migration and invasion of drug-resistant colon cancer cells

**Fig. 5:**
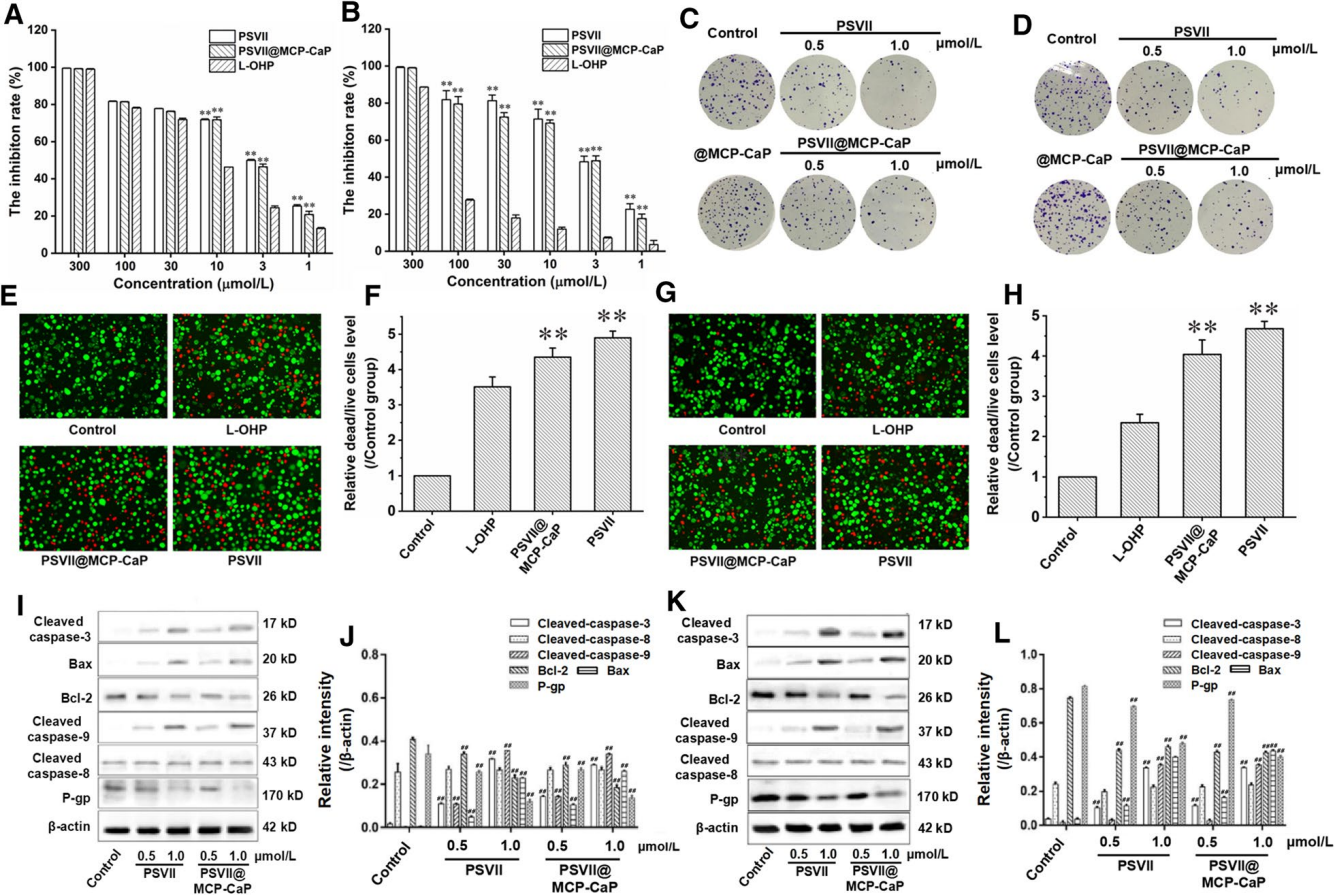
The inhibitory effect of PSVII@MCP-CaP on HCT116 cell and HCT116/L cell in vitro. **A** The inhibitory effect of PSVII@MCP-CaP on the proliferation of HCT116 cells. **B** The inhibitory effect of PSVII@MCP-CaP on the proliferation of HCT116/L cells. **C** The inhibitory effect of PSVII@MCP-CaP on the clone formation of HCT116 cell. **D** The inhibitory effect of PSVII@MCP-CaP on the clone formation of HCT116/L cell. **E** Live/dead cell staining of HCT116 cell. **F** Semi-quantitative statistical results of the ratio between dead HCT116 cell and live HCT116 cell. **G** Live/dead cell staining of HCT116/L cell. **H** Semi-quantitative statistical results of the ratio between dead HCT116/L cell and live HCT116/L cell. **I** The effect of PSVII@MCP-CaP on the expression of apoptosis-related proteins in HCT116 cell. **J** Semi-quantitative statistical results of the expression of apoptosis-related proteins in HCT116 cell. **K** The effect of PSVII@MCP-CaP on the expression of apoptosis-related proteins in HCT116/L cell. **L** Semi-quantitative statistical results of the expression of apoptosis-related proteins in HCT116/L cell. All data are expressed as mean ± SD (n = 3). **p < 0.01 vs L-OHP; ^##^*p* < 0.01 vs control

MTT assay indicated that oxaliplatin significantly inhibited the proliferation of HCT116 cells, HT-29 cell and SW620 cell, while its inhibitory effect on the proliferation of HCT116/L cells was greatly reduced, indicating that HCT116/L cells were resistant to oxaliplatin. Free PSVII and PSVII@MCP-CaP not only inhibited the proliferation of colon cancer cell (including HCT116 cells, HCT116/L cells, HT-29 cell and SW620 cell) in a concentration-dependent manner but also displayed a stronger inhibitory effect than oxaliplatin (Fig. 5A, B; Additional file 1: Fig. S5A, B). Besides, Clone formation experiment showed that in comparison with control group, @MCP-CaP had no significant effect on the clone formation of colon cancer cells, while the clone formation rate of HCT116 cells and HCT116/L cells in PSVII and PSVII@MCP-CaP treatment groups was significantly decreased. The higher the PSVII concentration was, the lower the clone formation rate was (Fig. 5C, D; Additional file 1: Fig. S6A, B). These results indicated that PSVII and PSVII@MCP-CaP inhibited the clone formation of colon cancer cells in a concentration-dependent manner. Furthermore, cell spheres growth experiment indicated that HCT116 cell spheres and HCT116/L cell spheres grew rapidly after treatment with PBS. However, the growth of cell spheres was markedly inhibited after treatment with PSVII and PSVII@MCP-CaP. On the 9th day after drug treatment, the volumes of HCT116 cell spheres and HCT116/L cell spheres in PSVII treatment group were reduced to 74.7% and 75.2% of the volume at the beginning of treatment. The volumes of HCT116 cell spheres and HCT116/L cell spheres treated by PSVII@MCP-CaP reduced to 51.5% and 56.5% of the volume at the beginning of treatment (Additional file 1: Fig. S6C, D). The above results demonstrated that PSVII@MCP-CaP inhibited the growth of HCT116 cell spheres and HCT116/L cell spheres. In addition, live/dead cell staining experiment showed that PSVII@MCP-CaP significantly increased the ratio between dead cell and live cell (Fig. 5E–H; Additional file 1: Fig. S5C–F), which was consistent with the results of MTT experiment.

The effects of PSVII@MCP-CaP on the expression of apoptosis-related proteins and P-gp in HCT116 cells and HCT116/L cells were investigated by western blot. The results showed that PSVII and PSVII@MCP-CaP increased Cleaved caspased-3, Cleaved caspased-9 and Bax protein expression, while they decreased Bcl-2 protein expression in HCT116 cells and HCT116/L cells in a concentration-dependent manner as compared to control group (Fig. 5I–L). Furthermore, PSVII and PSVII@MCP-CaP dose-dependently decreased the expression of P-gp in HCT116/L cells. These results demonstrated that PSVII@MCP-CaP promoted the apoptosis of HCT116 cells and HCT116/L cells through mitochondria-dependent pathway.

**Fig. 6:**
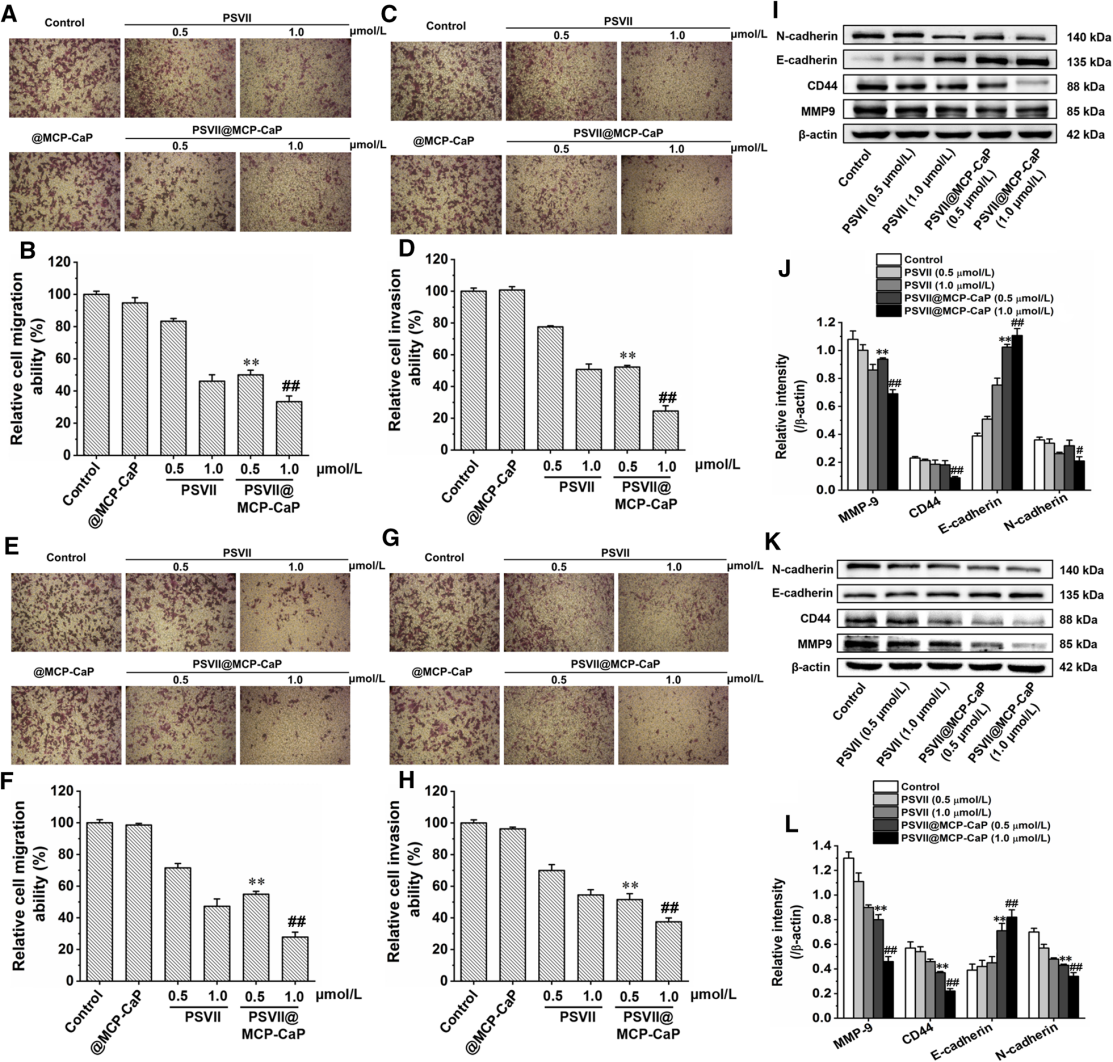
Effect of PSVII@MCP-CaP on the migration and invasion of HCT116 cell and HCT116/L cell. **A** Typical images of HCT116 cell migrated to transwell recipient chamber (× 200). **B** Semi-quantitative statistical results of HCT116 cell migration. **C** Typical images of HCT116 cell invaded to transwell recipient chamber (× 200). **D** Semi-quantitative statistical results of HCT116 cell invasion. **E** Typical images of HCT116/L cell migrated to transwell recipient chamber (× 200). **F** Semi-quantitative statistical results of HCT116/L cell migration. **G** Typical images of HCT116/L cell invaded to transwell recipient chamber (× 200). **H** Semi-quantitative statistical results of HCT116/L cell invasion. **I** Effect of PSVII@MCP-CaP on invasion-related proteins expression in HCT116 cell. **J** Semi-quantitative statistical results of invasion-related proteins expression in HCT116 cell. **K** Effect of PSVII@MCP-CaP on invasion-related proteins expression in HCT116/L cell. **L** Semi-quantitative statistical results of invasion-related proteins expression in HCT116/L cell. All data are expressed as mean ± SD (n = 3). **p < 0.01 vs PSVII (0.5 µmol/L); ^#^*p* < 0.05, ^##^*p* < 0.01 vs PSVII (1.0 µmol/L)

The migration and invasion experimental results indicated that in comparison with control group, @MCP-CaP had no significant effect on the migration and invasion rate of HCT116 cell, HCT116/L cell, HT-29 cell and SW620 cell. PSVII and PSVII@MCP-CaP significantly reduced the migration and invasion rate of HCT116 cell, HCT116/L cell, HT-29 cell and SW620 cell (Fig. 6A–H; Additional file 1: Fig. S7A–H). The higher the PSVII concentration was, the lower the migration and invasion rate was. These results demonstrated that PSVII and PSVII@MCP-CaP inhibited the migration and invasion of HCT116 cell, HCT116/L cell, HT-29 cell and SW620 cell in a concentration-dependent manner. Moreover, western blot results showed that protein expression of E-cadherin was higher in PSVII treated group and PSVII@MCP-CaP treated group than in the control group, while protein expression of MMP-9, CD44 and N-cadherin were significantly lower than in the control group (Fig. 6I–L; Additional file 1: Fig. S7I–L). These results indicated that PSVII and PSVII@MCP-CaP inhibited the migration and invasion of colon cancer cells through decreasing MMP-9, CD44 and N-cadherin protein expression.

### Biodistribution of PSVII@MCP-CaP in orthotopic drug-resistant colon cancer nude mice

**Fig. 7:**
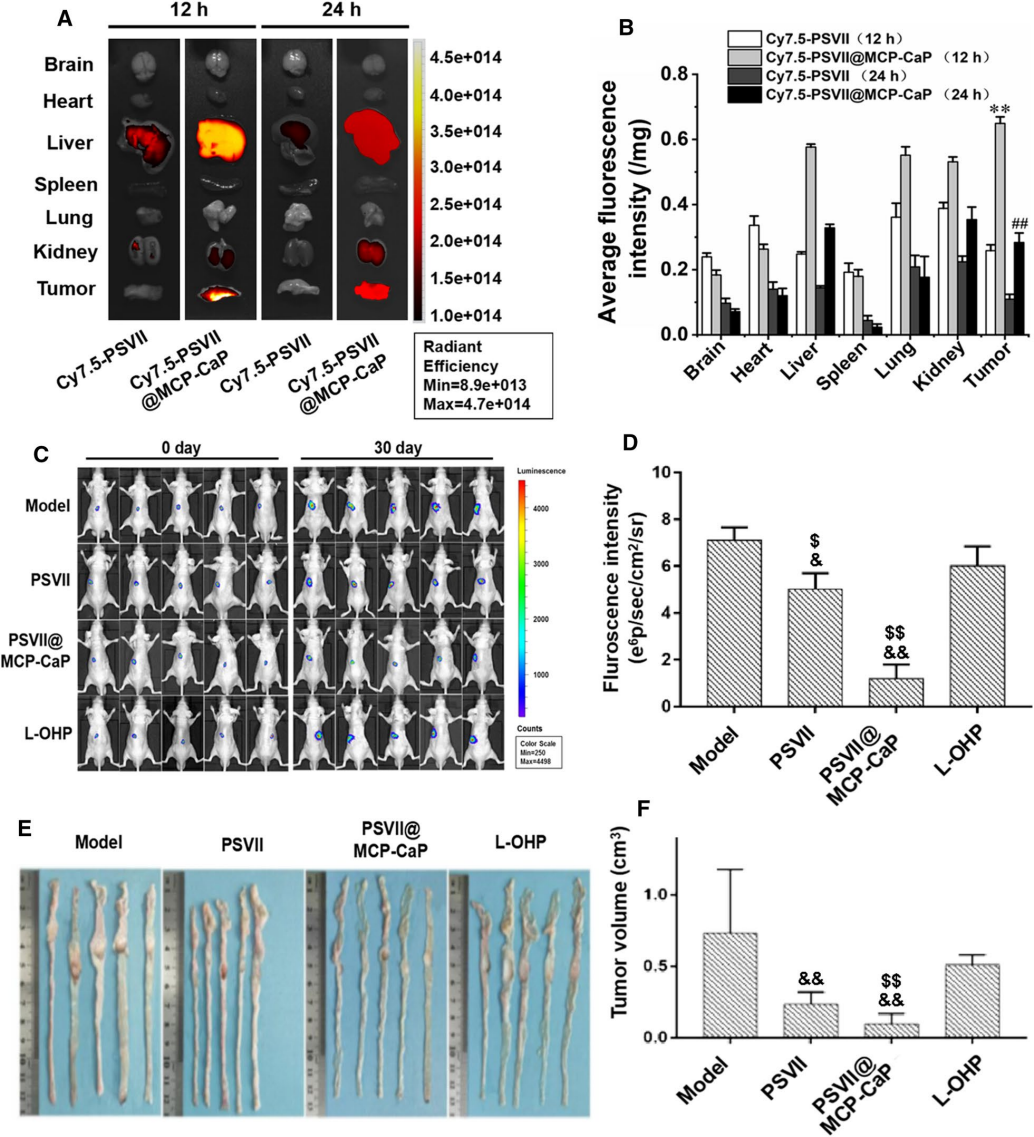
The distribution and anti-cancer activity of PSVII@MCP-CaP in orthotopic drug-resistant colon cancer nude mice. **A** Fluorescence distribution in various organs and cancer tissue of orthotopic colon cancer mice observed by in vivo bioluminescence imaging (n = 3). **B** The average relative fluorescence intensity in organs (fluorescence intensity/organ weight) detected by spectrofluorometer. **C** In vivo bioluminescence imaging of orthotopic drug-resistant colon cancer nude mice. **D** Semi-quantitative statistical results of fluorescence intensity of orthotopic drug-resistant colon cancer tissue. **E** Visual images of orthotopic drug-resistant colon cancer tissue. **F** Tumor volume of orthotopic drug-resistant colon cancer tissue. All data are expressed as means ± SD (n = 5). **p < 0.01 vs Cy7.5-PSVII (12 h); ^##^*p* < 0.01 vs Cy7.5-PSVII (24 h); ^&^*p* < 0.05, ^&&^*p* < 0.01 vs model; ^$^*p* < 0.05, ^$$^*p* < 0.01 vs L-OHP

After administration of Cy7.5-PSVII and Cy7.5-PSVII@MCP-CaP to nude mice with orthotopic drug-resistant colon cancer, fluorescence was mainly distributed in liver, kidney and tumor tissue. As compared with Cy7.5-PSVII, a large amount of fluorescence was mainly distributed in colon cancer tissue at 12 h and 24 h after Cy7.5-PSVII@MCP-CaP was administrated (Fig. 7A, B), indicating PSVII@MCP-CaP improved the targeting ability and retention time of PSVII to colon cancer tissue.

### In vivo anti orthotopic drug-resistant colon cancer activity of PSVII@MCP-CaP

On 31th day after drug administration, the mean fluorescence intensity in orthotopic drug-resistant colon cancer tissue showed no significant difference in oxaliplatin treated group and model group (Fig. 7C, D), suggesting that oxaliplatin had weak therapeutic effect on drug-resistant colon cancer. As compared with model group, the mean fluorescence intensity in orthotopic drug-resistant colon cancer tissue in PSVII@MCP-CaP treated group and PSVII treated group were significantly decreased, indicating that PSVII@MCP-CaP and PSVII significantly inhibited the growth of orthotopic drug-resistant colon cancer. Moreover, the inhibitory effect of PSVII@MCP-CaP on the growth of orthotopic drug-resistant colon cancer was stronger than that of PSVII. In addition, the images and tumor volume of orthotopic drug-resistant colon cancer tissue are showing in Fig. 7E, F. Compared with model group, PSVII@MCP-CaP and PSVII significantly reduced the tumor volume of orthotopic drug-resistant colon cancer tissue, which was consistent with the results observed by in vivo bioluminescence imager.

**Fig. 8:**
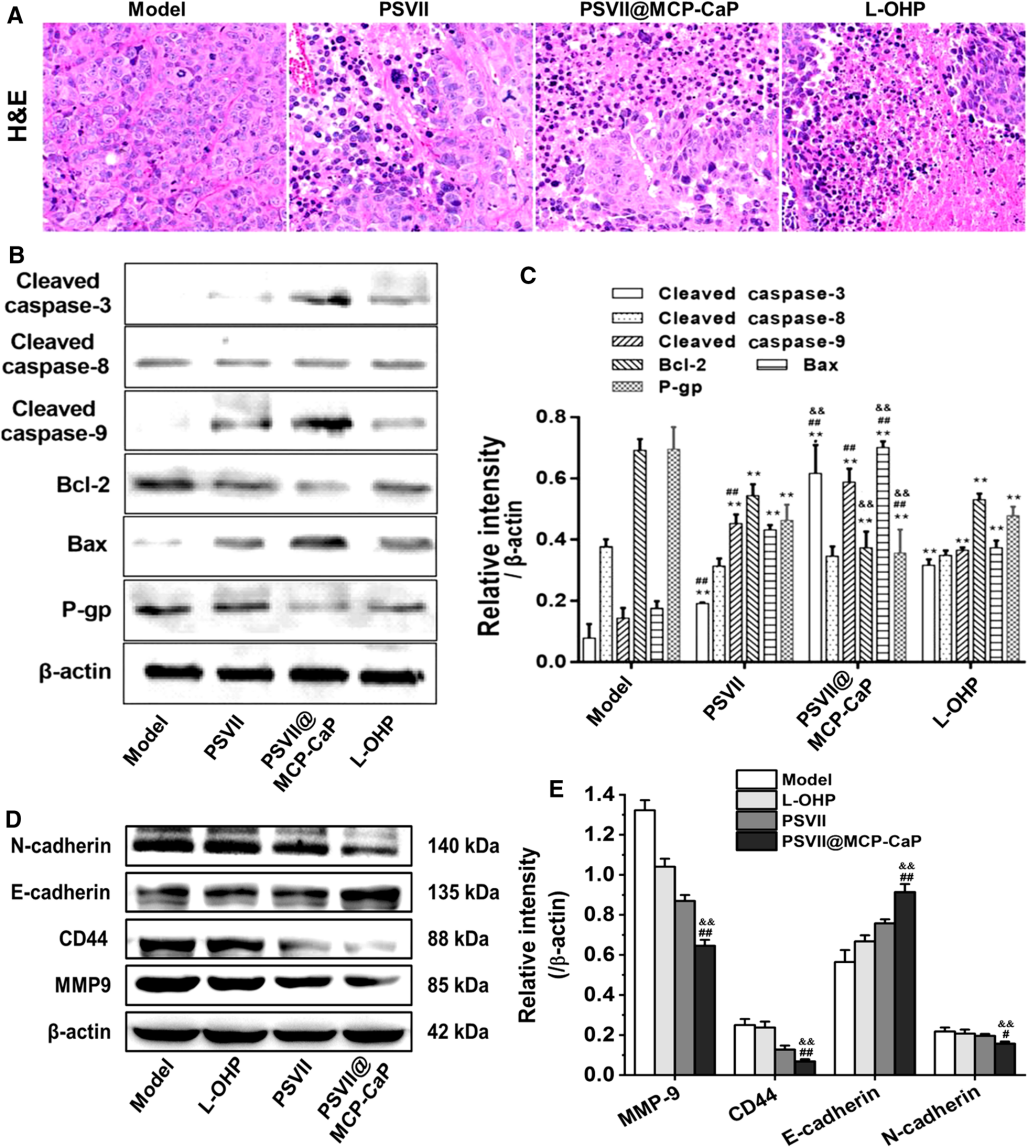
Effect of PSVII@MCP-CaP on tissue morphology and expression of apoptosis-related proteins and invasion-related proteins in orthotopic drug-resistant colon cancer tissue. **A** H&E staining of orthotopic drug-resistant colon cancer tissues (× 400). **B** Effect of PSVII@MCP-CaP on apoptosis-related proteins in orthotopic drug-resistant colon cancer tissue. **C** Semi-quantitative statistical results of apoptosis-related proteins expression in orthotopic drug-resistant colon cancer tissue. **D** Effect of PSVII@MCP-CaP on invasion-related proteins expression in orthotopic drug-resistant colon cancer tissue. **E** Semi-quantitative statistical results of invasion-related proteins expression in orthotopic drug-resistant colon cancer tissue. All data are expressed as mean ± SD (n = 3). **p < 0.01 vs model; ^#^*p* < 0.05, ^##^*p* < 0.01 vs L-OHP; ^&&^*p* < 0.01 vs PSVII

H&E staining of orthotopic drug-resistant colon cancer tissue showed that the nucleus was large and dense, and there was connective tissue in the stroma of drug-resistant colon cancer tissue in the model group. However, in oxaliplatin and PSVII treatment groups, nuclear pyknosis was found in some of drug-resistant colon cancer tissues. While in PSVII@MCP-CaP treatment group, there were obvious nuclear pyknosis and nucleus miniaturization in drug-resistant colon cancer tissues, and the number of cancer cells was significantly reduced (Fig. 8A). These results indicated that PSVII@MCP-CaP displayed significant toxicity on orthotopic drug-resistant colon cancer cells and improved the anti-tumor activity of PSVII.

### Effect of PSVII@MCP-CaP on expression of apoptosis-related and invasion-related proteins in orthotopic drug-resistant colon cancer tissue

Western blot results of orthotopic drug-resistant colon cancer tissues are showing in Fig. 8B, C. As compared with model group, the protein expressions of P-gp and Bcl-2 in orthotopic resistant colon cancer tissues were significantly decreased after treatment with PSVII and PSVII@MCP-CaP, and the protein expressions of Cleaved caspase-3, Cleaved caspase-9 and Bax were significantly increased. These results indicated that PSVII and PSVII@MCP-CaP induced apoptosis of drug-resistant colon cancer tissue and subsequently inhibited the growth of drug-resistant colon cancer through mitochondria-dependent programmed death pathway. The protein expressions of Cleaved caspase-3, Cleaved caspase-9 and Bax were significantly higher in PSVII@MCP-CaP treated group than in PSVII treated group, indicating PSVII@MCP-CaP displayed much stronger capability in inducing apoptosis of drug-resistant colon cancer tissue than that of PSVII. In addition, PSVII@MCP-CaP significantly reduced the protein expressions of CD44, MMP-9 and N-cadherin in drug-resistant colon cancer tissue, while the expression of E-cadherin was increased (Fig. 8D, E). These results demonstrated that PSVII@MCP-CaP inhibited the migration and invasion of colon cancer cells by decreasing the protein expression of CD44, MMP-9 and N-cadherin, while increasing the protein expression of E-cadherin.

### In vivo safety of PSVII@MCP-CaP

**Fig. 9:**
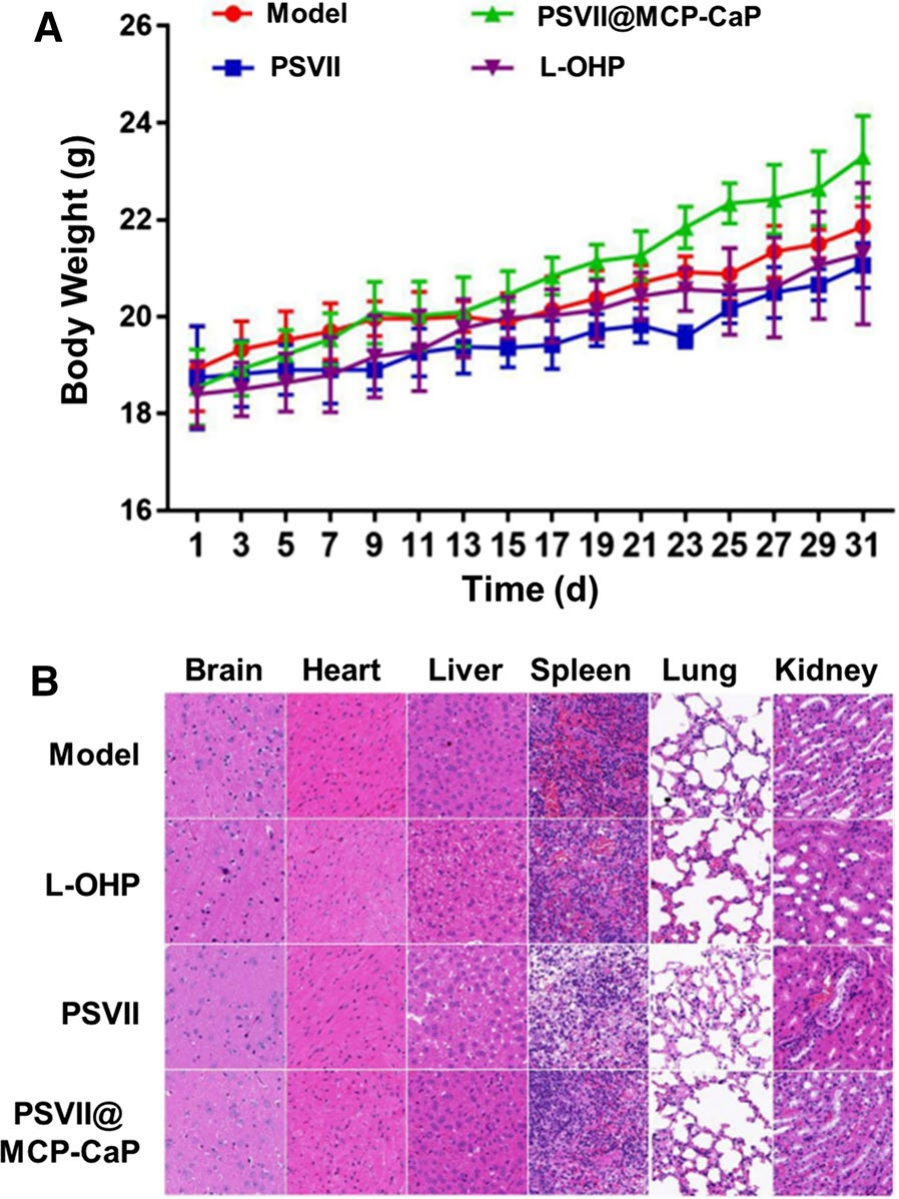
The safety evaluation of PSVII@MCP-CaP in cancer-bearing nude mice. **A** Body weight change of orthotopic drug-resistant colon cancer nude mice. **B** H&E staining of various tissues (× 400). All data are expressed as mean ± SD (n = 3)

The body weights of cancer-bearing nude mice are showing in Fig. 9A. As compared with model group, the body weight of cancer-bearing nude mice in PSVII@MCP-CaP treated groups showed no significant decline. H&E staining showed that there were vacuoles and granule degeneration in renal tubular epithelial cells of cancer-bearing nude mice in oxaliplatin treated mice (Fig. 9B), which indicated that oxaliplatin caused certain toxicity to kidney of cancer-bearing nude mice. Besides, the space of spleen tissue in PSVII treated group was enlarged and the nucleus size was irregular in cancer-bearing nude mice. There was granule degeneration of renal tubular epithelial cells in renal tissue. These results suggested that PSVII led to certain toxicity on spleen and kidney in cancer-bearing nude mice. There were not significant histopathological changes in organs in PSVII@MCP-CaP treated cancer-bearing nude mice.

## Discussion

PSVII displays strong anticancer activity. However, the structure of PSVII is similar to that of surfactants and has strong hemolytic activity and emulsification, which can rupture red blood cells and bring serious damage to human body. This greatly limits its clinical application. PSVII can bind to phospholipid bilayer on the erythrocyte membrane, resulting in changes in the permeability of the membrane [18–20]. This further leads to dilation and disintegration of red blood cells. Moreover, PSVII is insoluble compound both in water and fat. The molecular structure of PSVII does not contain functional groups binding calcium phosphate, so PSVII can’t be directly loaded into CaP nanoparticles by precipitation method in water. We found that CM-β-CD and PSVII could form inclusion complex in water, and CM-β-CD could bind calcium phosphate. Therefore, by using CM-β-CD and PSVII inclusion complex, PSVII could be embedded in CaP nanoparticles. This research explored a new method to prepare CaP nanoparticles loaded with a fat and water insoluble compound in water.

Finding new specific targets that can be recognized by drug delivery system is a hot and difficult topic in drug delivery system research. We found that Gal-3 was highly expressed on the membrane of colon cancer cells, which was consistent with previous reports [21, 22]. Immunofluorescence experimental results also indicated that a large amount of Gal-3 expressed in colon cancer tissue. Therefore, Gal-3 can be used as a specific binding target for colon cancer targeting drug delivery system [23, 24]. Furthermore, MCP is the hydrolyzed product of citrus pectin. The molecular weight and esterification degree of MCP are lower than that of citrus pectin. Previous studies have confirmed that MCP can block Gal-3 site on the surface of tumor cells, inhibiting the aggregation of cancer cells in blood circulation, and thus reducing cancer metastasis [25–27]. In addition, by blocking Gal-3 site, MCP inhibits cancer cells to take up nutrients from blood vessels, further slowing cancer tissue growth. Thus, MCP is a multi-function targeting ligand for colon cancer-specific drug delivery system.

The results of LSCM indicated that PSVII@MCP-CaP was taken up by drug-resistant colon cancer cells in time-dependent manner. When drug-resistant colon cancer cells were co-incubated with different concentrations of MCP, the uptake of PSVII@MCP-CaP by drug-resistant colon cancer cells was significantly reduced. In addition, the uptake experimental results also demonstrated that PSVII@MCP-CaP was taken up by HCT116/L cells mainly through clathrin mediated endocytosis. These results indicated that MCP mediated the uptake of PSVII@MCP-CaP in drug-resistant colon cancer cells. In vivo experimental results also showed that a large amount of PSVII@MCP-CaP accumulated in orthotopic drug-resistant colon cancer tissue, and PSVII@MCP-CaP penetrated into the deeper region of orthotopic drug-resistant colon cancer tissue. These results implied that PSVII@MCP-CaP delivered PSVII to drug-resistant colon cells in an active manner through interaction between Gal-3 and MCP.

CaP nanoparticle is an excellent drug delivery system with good safety. However, it is very difficult to prepare stabilized CaP nanoparticles. In order to improve the stability of CaP nanoparticles, researchers usually use chitosan, hyaluronic acid and other stabilizer during preparation of CaP nanoparticles in water, so as to inhibit the size growth of CaP nanoparticles and the transformation of CaP nanoparticles into calcium phosphate crystals [28, 29]. We have found that MCP contains a large number of carboxyl groups, which can bind with Ca^2+^ in calcium phosphate under alkaline conditions to increase the stability of CaP nanoparticles. The experimental results showed that MCP modified CaP nanoparticles (PSVII@MCP-CaP) could be stored stably in deionized water for 18 days.

There are two main apoptosis pathways, including death receptor-mediated apoptosis pathway and mitochondria-mediated apoptosis pathway [30, 31]. The death receptor pathway mainly promotes the production of caspase-8, and then activates the downstream caspase factor, thus initiating the apoptosis process dependent on the caspase-enzyme cascade [32]. Mitochondrial apoptosis mainly regulates the Bcl-2 protein family [33]. Studies have shown that the higher the Bcl-2/Bax ratio is, the stronger the mitochondrial membrane permeability became, which promotes the release of CytC, and then activates caspase-3 and finally causes cell apoptosis [34]. The experimental results indicated that PSVII@MCP-CaP reduced Bcl-2 expression and increased Bax, Cleaved caspased-9 and Cleaved caspased-3 expression in drug-resistant colon cancer tissue. These results demonstrated that PSVII@MCP-CaP promoted the apoptosis of drug-resistant colon cancer cells through mitochondria-mediated apoptosis pathway.

Local recurrence and metastasis are the main causes of death in colon cancer patients [35]. Studies have shown that E-cadherin is a key molecule to maintain the adhesion between cancer cells [36–38]. Inhibiting the expression of E-cadherin can reduce the adhesion between cancer cells and lead to the shedding of cancer cells from primary cancer tissue, which induces the metastasis of colon cancer [39, 40]. In addition, MMP-9 can degrade E-cadherin, subsequently reduce the adhesion between cancer cells [41, 42]. Finally, the invasion and metastasis of cancer cells are promoted. The experimental results showed that PSVII@MCP-CaP inhibited invasion and migration of drug-resistant colon cancer cells in a concentration-dependent manner. Further studies indicated that PSVII@MCP-CaP significantly increased the expression of E-cadherin and decreased the expression of N-cadherin and MMP-9 in drug-resistant colon cancer cells and orthotopic drug-resistant colon cancer tissue, which subsequently enhanced the adhesion between cancer cells in colon cancer tissue. Thus, PSVII@MCP-CaP inhibited the invasion and migration of drug-resistant colon cancer.

## Conclusion

PSVII@MCP-CaP significantly reduced the hemolysis effect of PSVII. PSVII was successfully encapsulated in CaP nanoparticles through formatting a CM-β-CD inclusion complex. By specific accumulating in orthotopic drug-resistant colon cancer tissue, PSVII@MCP-CaP effectively inhibited the growth of orthotopic drug-resistant colon cancer in nude mice. PSVII@MCP-CaP promoted the apoptosis of drug-resistant colon cancer cells through mitochondria-mediated apoptosis pathway. PSVII@MCP-CaP also significantly inhibited the invasion and migration of drug-resistant colon cancer cells by increasing the expression of E-cadherin and reducing the expression of N-cadherin and MMP-9.

## Supplementary Information

**Additional file 1: Figure S1** Immunofluorescence staining of Gal-3 in the colon tissue of normal nude mouse. Blue: nucleus; red: Gal-3 protein. **Figure S2.** XPS analysis of phosphorus (A), calcium (B), carbon (C), and oxygen (D) in PSVII@MCP-CaP. **Figure S3.** Thermogravimetric analysis of calcium phosphate powder (A), MCP (B), PSVII@CM-β-CD inclusion compound (C), @MCP-CaP (D), and PSVII@MCP-CaP (E). **Figure S4.** The cellular uptake mechanism of PSVII@MCP-CaP by HCT116/L cell. A The effect of uptake inhibitor on the uptake of PSVII@MCP-CaP by HCT116/L cell observed via LSCM. B Semi-quantitative analysis results of PSVII@MCP-CaP uptake by HCT116/L cell. All data are expressed as mean ± SD (n = 3). **p < 0.01 vs control. **Figure S5.** The inhibitory effect of PSVII@MCP-CaP on proliferation of HT-29 cell and SW620 cell in vitro. **A** The inhibitory effect of PSVII@MCP-CaP on the proliferation of HT-29 cell. **B** The inhibitory effect of PSVII@MCP-CaP on the proliferation of SW620 cell. **C** Live/dead cell staining of HT-29 cell. **D** Semi-quantitative statistical results of the ratio between dead HT-29 cell and live HT-29 cell. **E** Live/dead cell staining of SW620 cell. **F** Semi-quantitative statistical results of the ratio between dead SW620 cell and live SW620 cell. All data are expressed as mean ± SD (n = 5). **p < 0.01 vs L-OHP. **Figure S6. T**he statistical results of cell clone formation and growth of cell spheres.** A** Semi-quantitative statistical results of HCT116 cell clone formation. **B** Semi-quantitative statistical results of HCT116/L cell clone formation. **C** The effect of PSVII@MCP-CaP on the growth of HCT116 cell spheres. **D** The effect of PSVII@MCP-CaP on the growth of HCT116/L cell spheres. All data are expressed as mean ± SD (n = 3). **p < 0.01 vs control; ^##^*p* < 0.01 vs PBS. **Figure S7.** Effect of PSVII@MCP-CaP on the migration and invasion of HT-29 cell and SW620 cell. **A** Typical images of HT-29 cell migrated to transwell recipient chamber (× 200). **B** Semi-quantitative statistical results of HT-29 cell migration. **C** Typical images of HT-29 cell invaded to transwell recipient chamber (× 200). **D** Semi-quantitative statistical results of HT-29 cell invasion. **E** Typical images of SW620 cell migrated to transwell recipient chamber (× 200). **F** Semi-quantitative statistical results of SW620 cell migration. **G** Typical images of SW620 cell invaded to transwell recipient chamber (× 200). **H** Semi-quantitative statistical results of SW620 cell invasion. **I** Effect of PSVII@MCP-CaP on invasion-related proteins expression in HT-29 cell. **J** Semi-quantitative statistical results of invasion-related proteins expression in HT-29 cell. **K** Effect of PSVII@MCP-CaP on invasion-related proteins expression in SW620 cell. **L** Semi-quantitative statistical results of invasion-related proteins expression in SW620 cell. All data are expressed as means ± SD (n = 3). *p < 0.05, **p < 0.01 vs PSVII (0.5 µmol/L); ^#^*p* < 0.05, ^##^*p* < 0.01 vs PSVII (1.0 µmol/L)

## Data Availability

All data generated or analyzed during this study are included in this published article and its additional information file.

## Abbreviations

PSVII: Paris saponin VII
MCP: Modified citrus pectin
CaP: Calcium phosphate nanoparticles
ACP: Amorphous calcium phosphate
CM-β-CD: Carboxymethyl-β-cyclodextrin
Gal-3: Galectin-3
FBS: Foetal bovine serum
DAPI: 4ʹ,6-Diamidino-2-phenylindole dihydrochloride
L-OHP: Oxaliplatin
5-Fu: 5-Fluorouracil
MTT: 3-(4,5-Dimethylthiazol-2-yl)-2,5-diphenyltetrazolium bromide
HCT116/Fu cells: 5-Fu-resistant HCT116 cells
HCT116/L cells: L-OHP-resistant HCT116 cells
SDS-PAGE: Sulfate-polyacrylamide gel electrophoresis
PBS: Phosphate buffer saline
HR: Hemolysis rate
PDI: Polydispersity index
DTG: Derivative thermogravimetry
XPS: X-ray photoelectron spectrometer
LSCM: Laser scanning confocal microscopy
SEM: Scanning electron microscope
TEM: Transmission electron microscope
HPLC: High-performance liquid chromatography
