# Supplementary material for: MCP mediated active targeting calcium phosphate hybrid nanoparticles for the treatment of orthotopic drug-resistant colon cancer
Source: J Nanobiotechnology. 2021 Nov 17;19:367. doi: 10.1186/s12951-021-01115-9 (PMC8600743; doi:10.1186/s12951-021-01115-9)
Supplement: Supplementary file 1 — Additional file 1: Figure S1 Immunofluorescence staining of Gal-3 in the colon tissue of normal nude mouse. Blue: nucleus; red: Gal-3 protein. Figure S2. XPS analysis of phosphorus (A), calcium (B), carbon (C), and oxygen (D) in PSVII@MCP-CaP. Figure S3. Thermogravimetric analysis of calcium phosphate powder (A), MCP (B), PSVII@CM-β-CD inclusion compound (C), @MCP-CaP (D), and PSVII@MCP-CaP (E). Figure S4. The cellular uptake mechanism of PSVII@MCP-CaP by HCT116/L cell. A The effect of uptake inhibitor on the uptake of PSVII@MCP-CaP by HCT116/L cell observed via LSCM. B Semi-quantitative analysis results of PSVII@MCP-CaP uptake by HCT116/L cell. All data are expressed as mean ± SD (n = 3). **p < 0.01 vs control. Figure S5. The inhibitory effect of PSVII@MCP-CaP on proliferation of HT-29 cell and SW620 cell in vitro. A The inhibitory effect of PSVII@MCP-CaP on the proliferation of HT-29 cell. B The inhibitory effect of PSVII@MCP-CaP on the proliferation of SW620 cell. C Live/dead cell staining of HT-29 cell. D Semi-quantitative statistical results of the ratio between dead HT-29 cell and live HT-29 cell. E Live/dead cell staining of SW620 cell. F Semi-quantitative statistical results of the ratio between dead SW620 cell and live SW620 cell. All data are expressed as mean ± SD (n = 5). **p < 0.01 vs L-OHP. Figure S6. The statistical results of cell clone formation and growth of cell spheres. A Semi-quantitative statistical results of HCT116 cell clone formation. B Semi-quantitative statistical results of HCT116/L cell clone formation. C The effect of PSVII@MCP-CaP on the growth of HCT116 cell spheres. D The effect of PSVII@MCP-CaP on the growth of HCT116/L cell spheres. All data are expressed as mean ± SD (n = 3). **p < 0.01 vs control; ##p < 0.01 vs PBS. Figure S7. Effect of PSVII@MCP-CaP on the migration and invasion of HT-29 cell and SW620 cell. A Typical images of HT-29 cell migrated to transwell recipient chamber (× 200). B Semi-quantitative statistical results o [file 12951_2021_1115_MOESM1_ESM.docx]

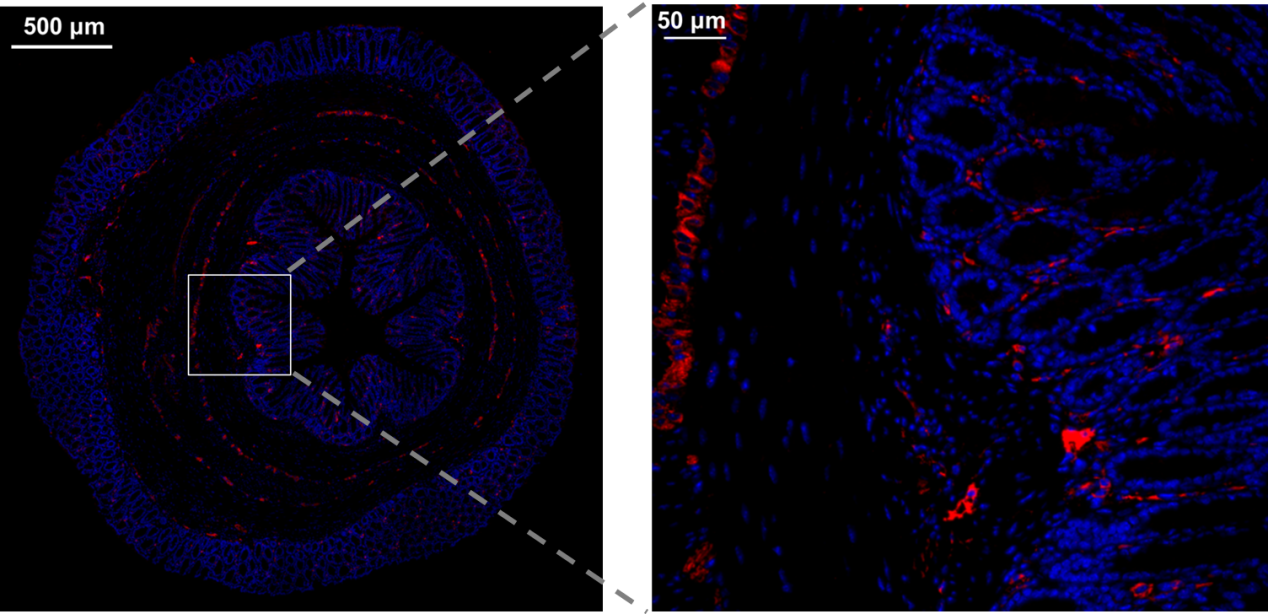


**Figure S1** Immunofluorescence staining of Gal-3 in the colon tissue of normal nude mouse. Blue: nucleus; red: Gal-3 protein


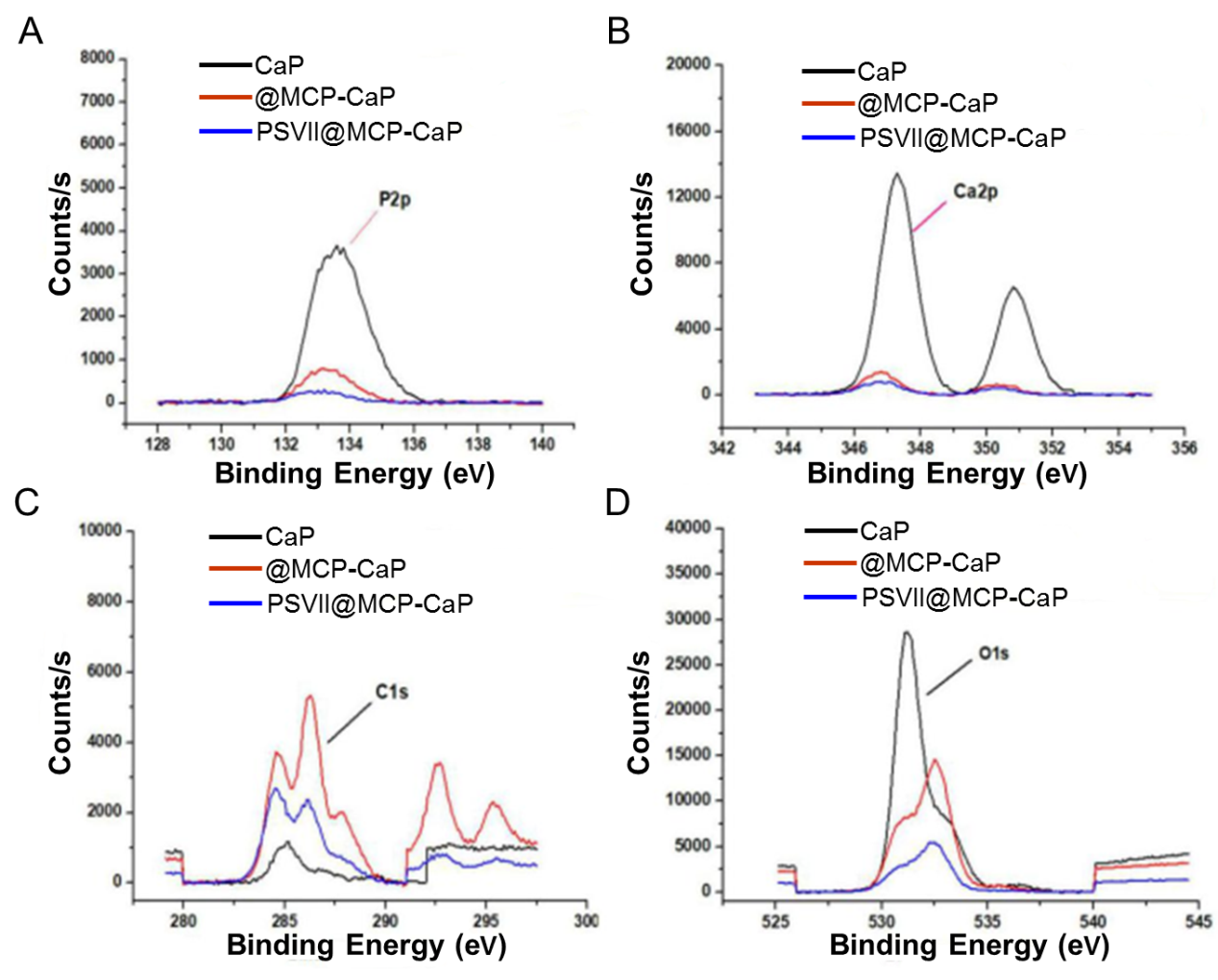


**Figure S2** XPS analysis of phosphorus (A), calcium (B), carbon (C), and oxygen (D) in PSVII@MCP-CaP


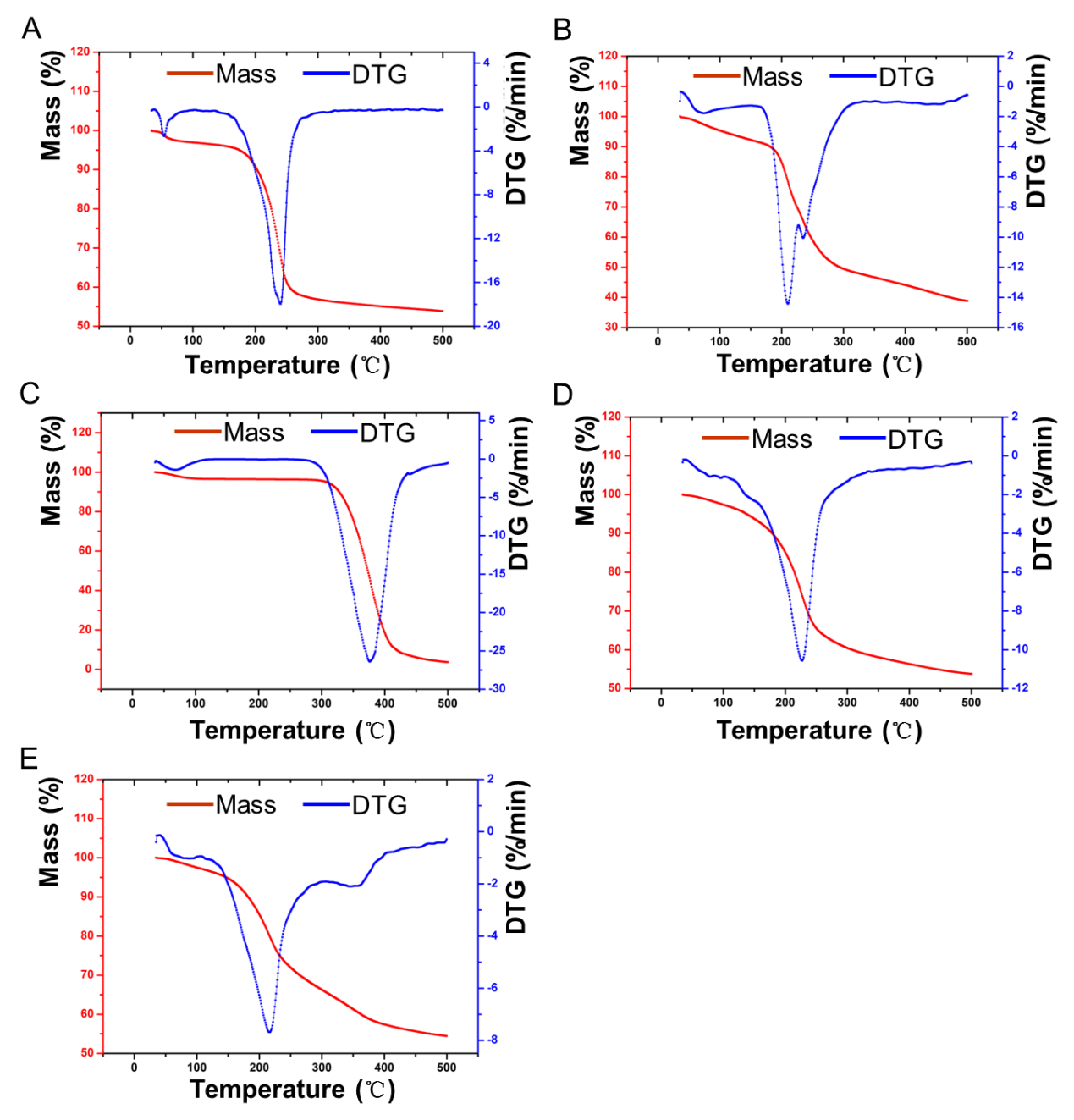


**Figure S3** Thermogravimetric analysis of calcium phosphate powder (A), MCP (B), PSVII@CM-β-CD inclusion compound (C), @MCP-CaP (D), and PSVII@MCP-CaP (E)


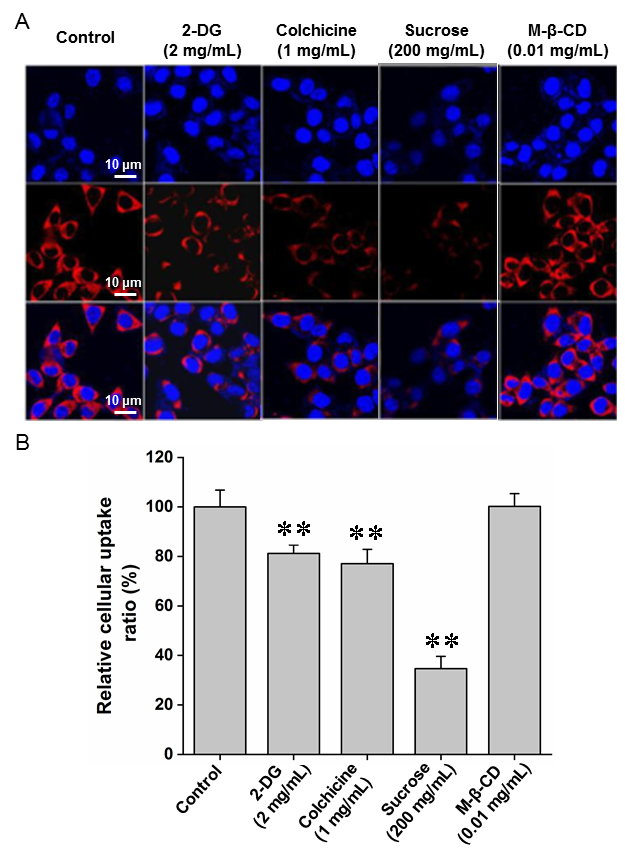


**Figure S4** The cellular uptake mechanism of PSVII@MCP-CaP by HCT116/L cell. A The effect of uptake inhibitor on the uptake of PSVII@MCP-CaP by HCT116/L cell observed *via* LSCM. B Semi-quantitative analysis results of PSVII@MCP-CaP uptake by HCT116/L cell. All data are expressed as mean±SD (n=3). ^**^*p* < 0.01 vs control

**
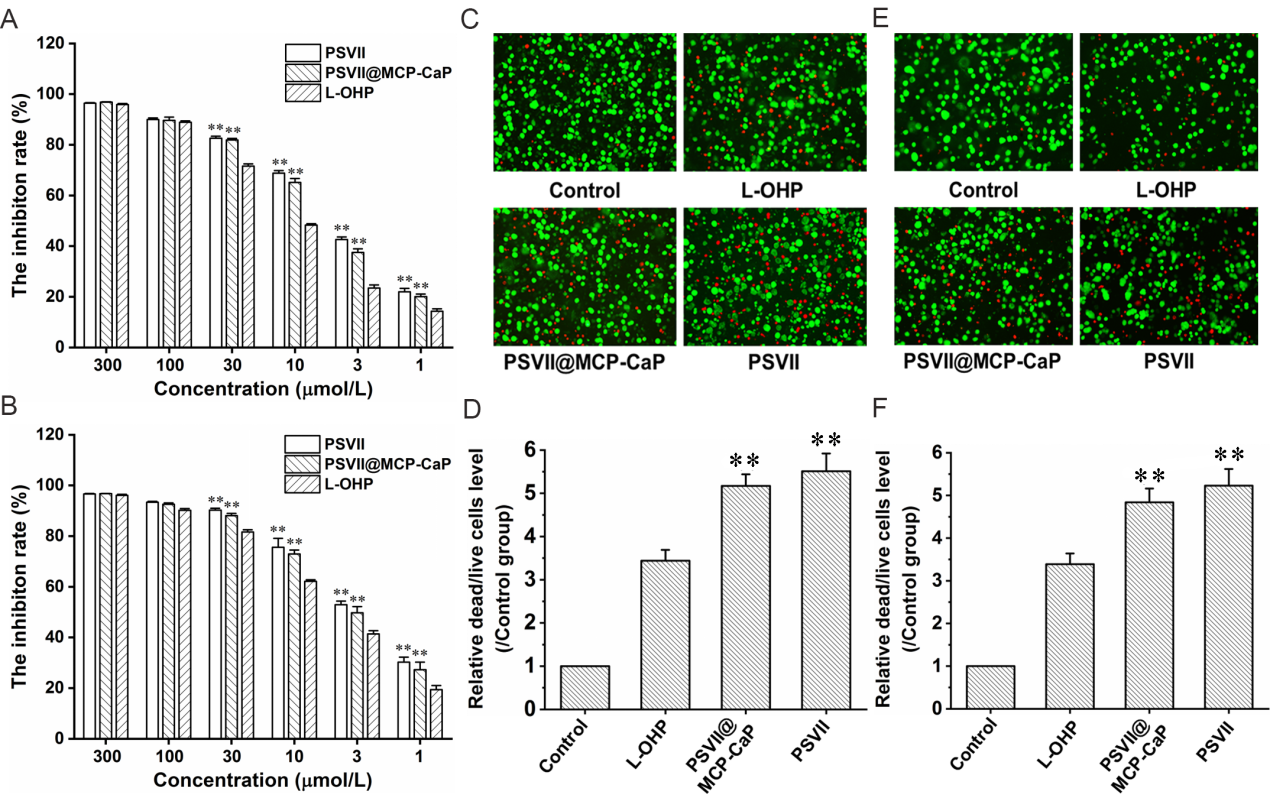
**

**Figure S5** The inhibitory effect of PSVII@MCP-CaP on proliferation of HT-29 cell and SW620 cell *in vitro*. **A** The inhibitory effect of PSVII@MCP-CaP on the proliferation of HT-29 cell. **B** The inhibitory effect of PSVII@MCP-CaP on the proliferation of SW620 cell. **C** Live/dead cell staining of HT-29 cell. **D** Semi-quantitative statistical results of the ratio between dead HT-29 cell and live HT-29 cell. **E** Live/dead cell staining of SW620 cell. **F** Semi-quantitative statistical results of the ratio between dead SW620 cell and live SW620 cell. All data are expressed as mean±SD (n=5). ^**^*p* < 0.01 vs L-OHP


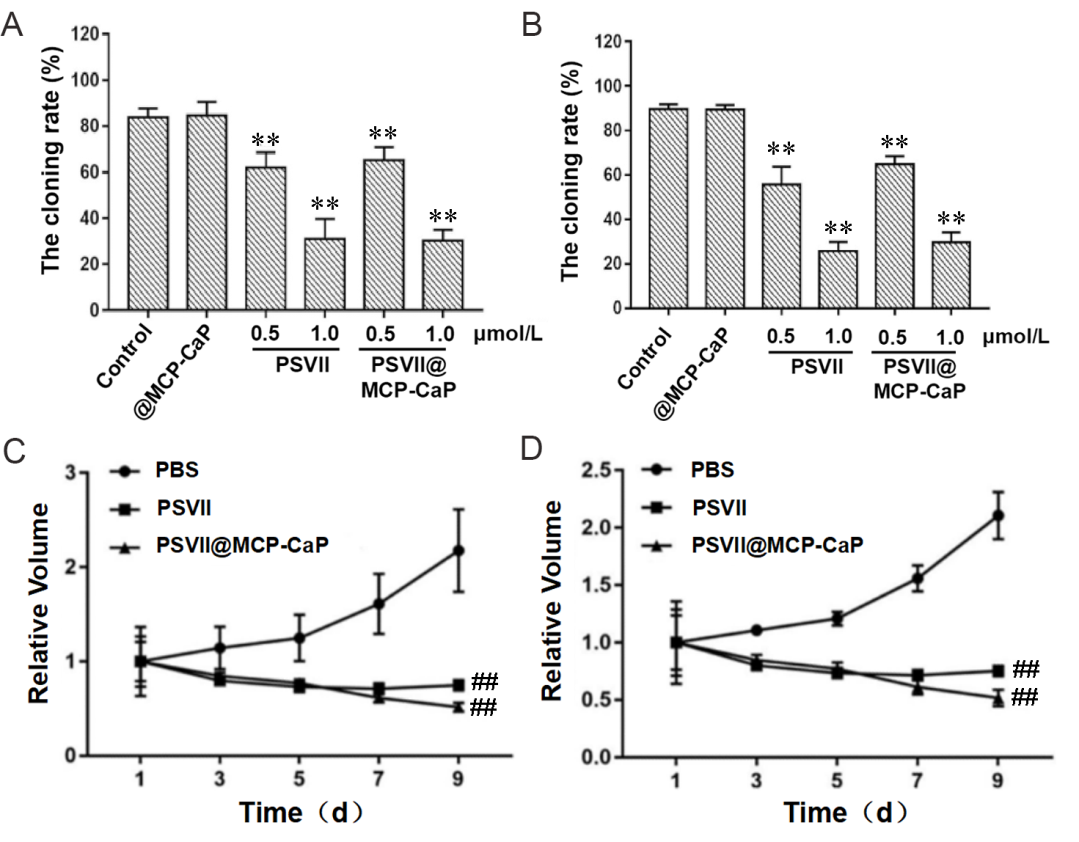


**Figure S6. T**he statistical results of cell clone formation and growth of cell spheres. **A** Semi-quantitative statistical results of HCT116 cell clone formation. **B** Semi-quantitative statistical results of HCT116/L cell clone formation. **C** The effect of PSVII@MCP-CaP on the growth of HCT116 cell spheres. **D** The effect of PSVII@MCP-CaP on the growth of HCT116/L cell spheres. All data are expressed as mean±SD (n=3). ^**^*p* < 0.01 vs control; ^##^*p* < 0.01 vs PBS


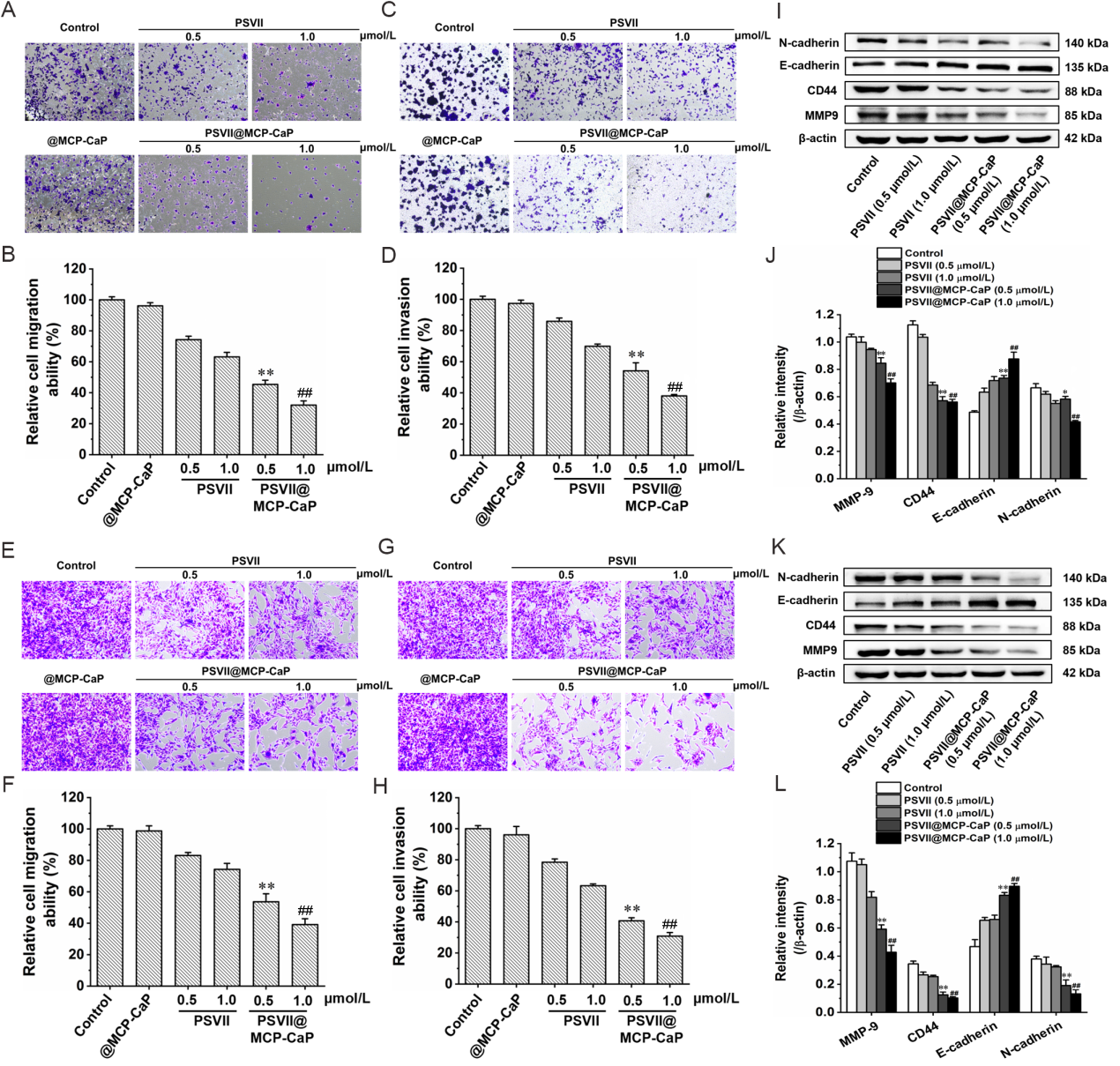


**Figure S7.** Effect of PSVII@MCP-CaP on the migration and invasion of HT-29 cell and SW620 cell. **A** Typical images of HT-29 cell migrated to transwell recipient chamber (×200). **B** Semi-quantitative statistical results of HT-29 cell migration. **C** Typical images of HT-29 cell invaded to transwell recipient chamber (×200). **D** Semi-quantitative statistical results of HT-29 cell invasion. **E** Typical images of SW620 cell migrated to transwell recipient chamber (×200). **F** Semi-quantitative statistical results of SW620 cell migration. **G** Typical images of SW620 cell invaded to transwell recipient chamber (×200). **H** Semi-quantitative statistical results of SW620 cell invasion. **I** Effect of PSVII@MCP-CaP on invasion-related proteins expression in HT-29 cell. **J** Semi-quantitative statistical results of invasion-related proteins expression in HT-29 cell. **K** Effect of PSVII@MCP-CaP on invasion-related proteins expression in SW620 cell. **L** Semi-quantitative statistical results of invasion-related proteins expression in SW620 cell. All data are expressed as means±SD (n=3). ^*^*p* < 0.05, ^**^*p* < 0.01 vs PSVII (0.5 µmol/L); ^#^*p* < 0.05, ^##^*p* < 0.01 vs PSVII (1.0 µmol/L)
